# Integration of molecular profiles in a longitudinal wellness profiling cohort

**DOI:** 10.1038/s41467-020-18148-7

**Published:** 2020-09-08

**Authors:** Abdellah Tebani, Anders Gummesson, Wen Zhong, Ina Schuppe Koistinen, Tadepally Lakshmikanth, Lisa M. Olsson, Fredrik Boulund, Maja Neiman, Hans Stenlund, Cecilia Hellström, Max J. Karlsson, Muhammad Arif, Tea Dodig-Crnković, Adil Mardinoglu, Sunjae Lee, Cheng Zhang, Yang Chen, Axel Olin, Jaromir Mikes, Hanna Danielsson, Kalle von Feilitzen, Per-Anders Jansson, Oskar Angerås, Mikael Huss, Sanela Kjellqvist, Jacob Odeberg, Fredrik Edfors, Valentina Tremaroli, Björn Forsström, Jochen M. Schwenk, Peter Nilsson, Thomas Moritz, Fredrik Bäckhed, Lars Engstrand, Petter Brodin, Göran Bergström, Mathias Uhlen, Linn Fagerberg

**Affiliations:** 1 Science for Life Laboratory, Department of Protein Science, KTH Royal Institute of Technology, Stockholm, Sweden; 2 Wallenberg Laboratory and Sahlgrenska Center for Cardiovascular and Metabolic Research, Department of Molecular and Clinical Medicine, Institute of Medicine, University of Gothenburg, Gothenburg, Sweden; 3 Region Västra Götaland, Sahlgrenska University Hospital, Department of Clinical Genetics and Genomics, Gothenburg, Sweden; 4 Center for Translational Microbiome Research, Department of Microbiology, Tumor and Cell Biology, Karolinska Institutet, Stockholm, Sweden; 5 Science for Life Laboratory, Department of Women’s and Children’s Health, Karolinska Institutet, Stockholm, Sweden; 6 Swedish Metabolomics Centre, Department of Molecular Biology, Umeå University, 901 87 Umeå, Sweden; 7 Centre for Host-Microbiome Interactions, Faculty of Dentistry, Oral & Craniofacial Sciences, King’s College London, London, UK; 8 Region Västra Götaland, Sahlgrenska University Hospital, Department of Internal Medicine, Gothenburg, Sweden; 9 Department of Molecular and Clinical Medicine, Institute of Medicine, University of Gothenburg, Gothenburg, Sweden; 10 Region Västra Götaland, Sahlgrenska University Hospital, Department of Cardiology, Gothenburg, Sweden; 11 Codon Consulting, 118 26 Stockholm, Sweden; 12 Department of Learning, Informatics, Management and Ethics, Karolinska Institutet, Stockholm, Sweden; 13 Department of Medical Epidemiology and Biostatistics, Karolinska Institutet, Stockholm, Sweden; 14 Swedish Metabolomics Centre, Department of Forest Genetics and Plant Physiology, Swedish University of Agricultural Sciences, 907 36 Umeå, Sweden; 15 Region Västra Götaland, Sahlgrenska University Hospital, Department of Clinical Physiology, Gothenburg, Sweden; 16 Novo Nordisk Foundation Center for Basic Metabolic Research, Section for Metabolic Receptology and Enteroendocrinology, Faculty of Health Sciences, University of Copenhagen, Copenhagen, Denmark; 17 Center for Biosustainability, Danish Technical University, Copenhagen, Denmark

**Keywords:** Computational biology and bioinformatics, Data integration, Molecular medicine

## Abstract

An important aspect of precision medicine is to probe the stability in molecular profiles among healthy individuals over time. Here, we sample a longitudinal wellness cohort with 100 healthy individuals and analyze blood molecular profiles including proteomics, transcriptomics, lipidomics, metabolomics, autoantibodies and immune cell profiling, complemented with gut microbiota composition and routine clinical chemistry. Overall, our results show high variation between individuals across different molecular readouts, while the intra-individual baseline variation is low. The analyses show that each individual has a unique and stable plasma protein profile throughout the study period and that many individuals also show distinct profiles with regards to the other omics datasets, with strong underlying connections between the blood proteome and the clinical chemistry parameters. In conclusion, the results support an individual-based definition of health and show that comprehensive omics profiling in a longitudinal manner is a path forward for precision medicine.

## Abstract (web summary)

An important aspect of precision medicine is to probe the stability in molecular profiles among healthy individuals over time. Here, the authors sample a longitudinal wellness cohort and analyse blood molecular profiles as well as gut microbiota composition.

## Introduction

A challenge for the field of precision medicine is defining a healthy population to estimate the normal range of clinical parameters across population strata^1^. The dawn of many new omics tools for analyzing clinical samples such as genomics, proteomics and metabolomics has opened up new possibilities to study both health and disease with high throughput along with high analytical precision and clinical accuracy^2,3^ and projects with broad analytical breadth have been initiated including the Pioneer 100 Wellness Project^4^, multi-omics profiling of the blood and gut microbiota to explore weight gain and loss^5^, omics-based biological phenotyping to probe individualized aging^6,7^, precision medicine directed integration of whole genome sequencing combined with imaging^8^, and a generation of a genome atlas for the human plasma proteome^9^. In this way, personal omics profiles, composed of the genomics, transcriptomics, proteomics, metabolomics, and fecal microbiota, could be defined and used to monitor drug interventions. Molecular profiling has also been the basis of the athlete biological passport used by the World Antidoping Association for an individualized longitudinal monitoring of elite athletes^10–14^. These studies demonstrate the importance of deep profiling of healthy individuals as a fundamental aspect of precision medicine by probing the underlying high biological plasticity. However, more in-depth studies are needed to define the wellness parameters of individuals and to analyze the variability among omics data between individuals as well as within an individual overtime.

Here, we describe a wellness profiling study of 101 individuals between 50 and 65 years old, based on a combination of classical clinical chemistry, advanced medical imaging and extensive omics profiling, including the analysis of the plasma proteome, the plasma metabolome, blood cell composition (immune cytome), transcriptome, autoantibody reactivity profiles, and gut microbiota composition. In this study, the main objective is to allow for a high biological and clinical data granularity based on molecular depth with regards to the number of targets analyzed in a sensitive manner. However, it is important to validate the results by follow-up studies in which the most interesting targets is analyzed in larger patient cohorts. Our aim is to probe the uniqueness and stability of an individual’s molecular profiles during a 2-year period by sampling the participants repeatedly at six different time points and thereby investigating the relationships between omics profiles and classical routine clinical chemistry measurements. We find that each individual carries a unique, and stable, molecular profile that is perturbed globally by changes in lifestyle and more transiently by infectious disease. This lays the foundation for future precision medicine based on the longitudinal monitoring of wellness.

## Results

### The study design

**Fig. 1:**
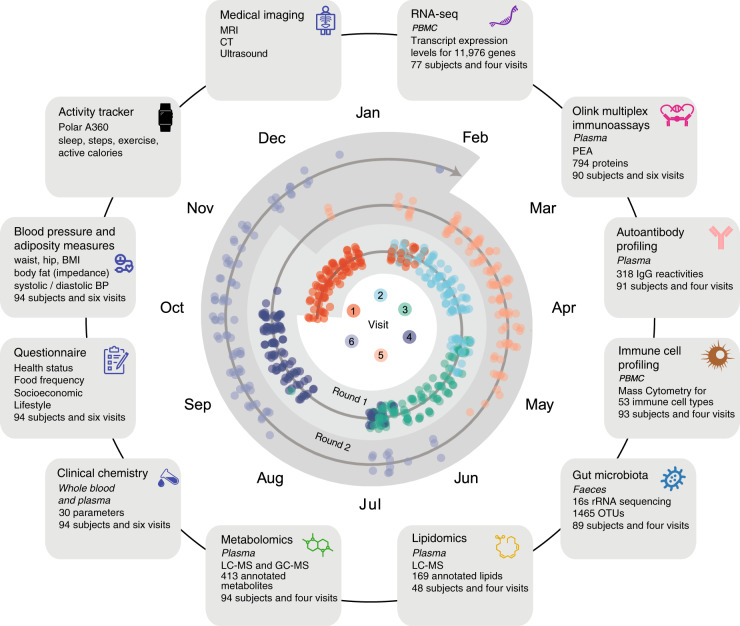
Overview of the S3WP program. The outer part represents all types of data that have been analyzed for this study. The inner part shows the distribution across the months of a year for all visits for each of the 94 subjects that completed the program, where round one includes visits one to four with approximately three months intervals and round two includes visits 5 and 6 with ~6 months intervals. PBMC peripheral blood mononuclear cell; PEA Proximity Extension Assay; OTUs operational taxonomic units; IgG Immunoglobulin G; rRNA Ribosomal Ribonucleic Acid; LC–MS liquid chromatography–mass spectrometry; GC–MS gas chromatography–mass spectrometry; BP blood pressure; BMI body mass index; MRI Magnetic resonance imaging; CT computed tomography.

The Swedish SciLifeLab SCAPIS Wellness Profiling (S3WP) program presented here is based on the SCAPIS study^15^ and consists of a smaller cohort of 101 individuals between 50 and 65 years old, enrolled from the large population and followed longitudinally for 2 years with repeated analyses of molecular markers in blood and stool samples in combination with physical measurements. Of the 101 subjects that were first included, 99 completed the 4 study visits of the first round with 3 months’ intervals, and 94 completed the 2 visits of the second round sampled at approximately six-months intervals (Fig. 1). A summary of the subject characteristics is shown in Table S1. All collected samples were analyzed by a comprehensive set of platforms including plasma proteome analysis based on proximity extension assay, IgG autoantibody reactivity profiling, immune cell profiling based on mass cytometry, transcriptomics profiling of Peripheral Blood Mononuclear Cells (PBMCs) based on RNA-seq, gut microbiota analyses using 16S rRNA gene profiling and plasma metabolite and lipids profiling using LC–MS (Fig. 1). In addition to these molecular profiles and extensive clinical assessment, each individual was also followed using detailed questionnaires at every visit and tracking devices that monitor physical activity levels and sleep patterns. For the clinical chemistry and the protein profiling, we included data for the complete two rounds with a total of six analyzed visits, whereas the other datasets consist of data for four visits sampled during the first round.

### Clinical chemistry and anthropometric measurements

**Fig. 2:**
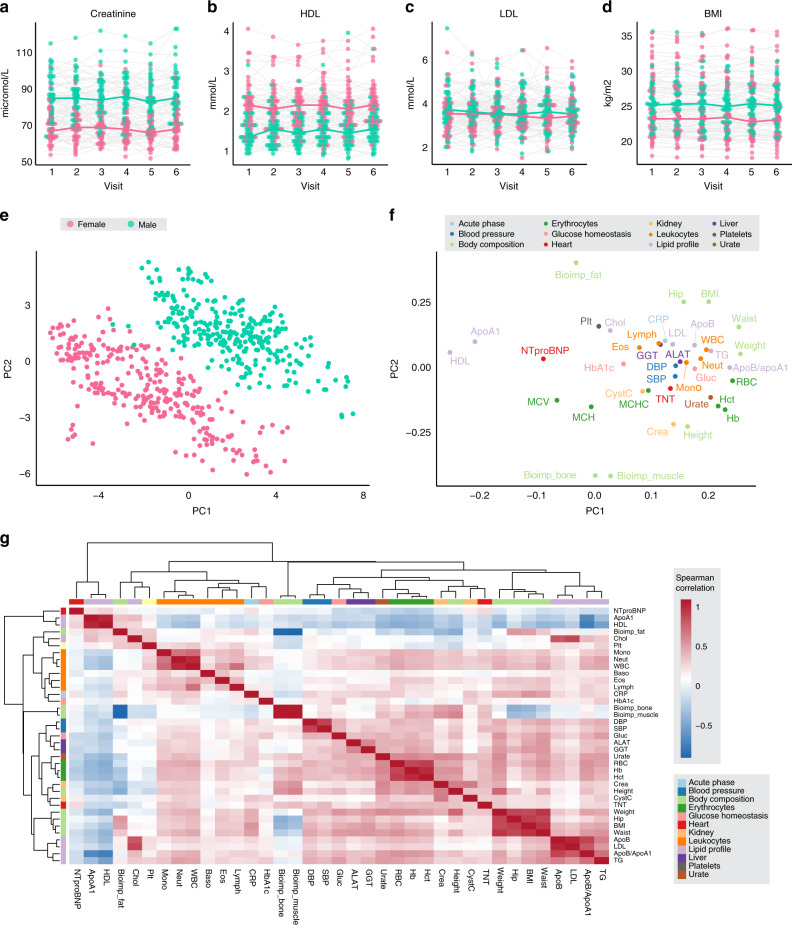
Clinical chemistry and anthropometrics longitudinal variation. Clinical chemistry variation across the six visits for 94 subjects in: **a** Creatinine, **b** high-density lipoproteins (HDL), **c** low-density lipoproteins (LDL), as well as the variation in **d** body mass index (BMI). The color indicates males and females, the colored lines visualize the medians for each visit and sex respectively, and each individual is connected by a gray line. Principal Component Analysis (PCA) based on data from visits 1–6 for 41 parameters including anthropometrics, clinical chemistry and hematology variables is visualized as **e** scores plot showing clear separation between males and females and **f** loadings plot showing the relationship between the underlying variables (Explained variance: PC1 = 23%, PC 2 = 13%). **g** Heatmap showing the pairwise Spearman correlations between 37 clinical chemistry and anthropometry variables.

We investigated a total of 30 clinical chemistry parameters and three selected examples are shown in Fig. 2a–c to highlight the longitudinal variation across the visits. Creatinine is a routinely used kidney marker and it is known that its levels (Fig. 2a) are notably higher in males compared to females due to sex differences in muscle mass^16^, and this is also observed in our data. The results show stable creatinine levels in all individuals indicating a stable renal function during the study period. The results of high-density lipoprotein (HDL) (Fig. 2b) also show, as expected, clear sex-specific trends^17^ with higher levels in females than males, in contrast to low-density lipoprotein (LDL) (Fig. 2c), which is more variable across the six visits. In addition, we analyzed anthropometrics, such as weight, height, diastolic/systolic blood pressure, and calculated the body mass index (BMI). The average BMI is also stable overtime with an average BMI of 25.3 for visit one and 25.1 for visit six although there are some deviations at the individual level (Fig. 2d). Similar plots for another 14 of the assessed variables are shown in Supplementary Fig. 1 and with animated versions in Supplementary Movie 1. The mean body weight remained stable during the observation period, with the exception of one subject experiencing marked diet-induced weight loss, −15.8 kg (−34.8 pounds) between visits three and four. The clinical chemistry variables were largely stationary at the group level and the most noticeable finding at the individual level was a pronounced CRP elevation (79.0 mg/L) at visit 2 in one subject. A summary of all longitudinally collected clinical variables including classification and a list of abbreviations is given in Supplementary Dataset 1.

Principal Component Analysis (PCA)^18^ is used to assess the general trends in the data and the resulting score plot shows a clear separation between males and females based on their clinical chemistry and anthropometrics data (Fig. 2e). In the loading plot (Fig. 2f), the interrelationships between all variables are summarized and the main parameters explaining this difference include anthropometrics, hemoglobin (Hb), creatinine, red blood cell (RBC) count, HDL, Apolipoprotein A1(ApoA1) and hematocrit parameters. Hierarchical clustering was performed based on the correlation between a selection of clinical variables and anthropometrics parameters and the pairwise relationship between all variables is visualized in Fig. 2g, based on mean values, whereas the correlation based on separate visits are shown in Supplementary Fig. 2, implying high correlation and longitudinal stability for most clinical variables.

### Longitudinal molecular profiling

The primary objective of this study is to analyze the global molecular omics profiles of each of the individuals and to investigate the stability of such profiles during the study period. The autoantibody profile of each individual was analyzed as previously described^19^ using a bead-based protein array based on recombinant human proteins generated within the framework of the Human Protein Atlas^20^ to analyze the plasma IgG reactivity toward 318 human protein fragments for 91 individuals. For the plasma proteome analysis, eleven panels of antibody-based, multiplex assays^21^ toward human blood proteins were included. The result from the proximity extension assay technology allowed the quantitative analysis of altogether 794 target proteins for 90 individuals. The PBMC immune cell profiles were characterized by mass cytometry using a 38-parameter panel covering all major immune cell populations. Out of these, the 53 most widely abundant and robustly detected cell populations were included in the analysis of 93 individuals. The metabolites in plasma were also determined using a LC–MS based metabolomics analysis^22^ including 413 annotated metabolites for 94 individuals. The plasma lipidome was determined using a LC–MS based lipidomics analysis detecting altogether 169 annotated lipids for 48 of the individuals. The gut microbiota of all the individuals was analyzed based on 16S rRNA gene sequencing^23^ and resulted in data for 89 individuals from 1465 operational taxonomic units (OTUs). Finally, the PBMC transcriptome was assessed using RNA-seq, resulting in gene expression data for 19,670 protein-coding genes, out of which 11,976 were detected in the 77 analyzed individuals. The complete list of all analyzed variables per dataset is available in Supplementary Dataset 2.

### Analyzing the individual longitudinal stability and variability

**Fig. 3:**
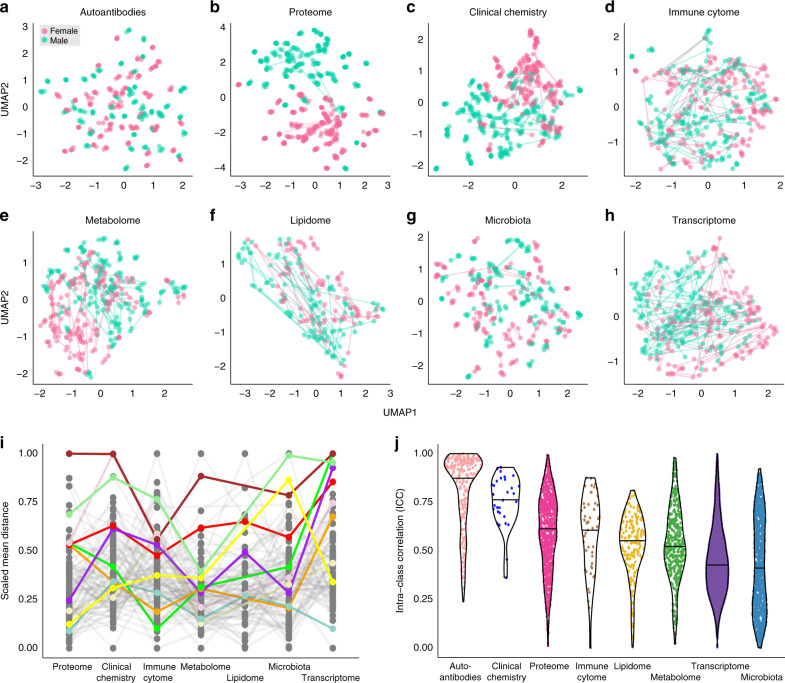
Global molecular profiles and the individual variation. The global profiles across visits were analyzed using UMAP for eight different datasets used in the study: **a** autoantibodies (*n* = 318) analyzed in plasma and based on 91 subjects; **b** plasma protein expression data for 794 proteins in 90 subjects; **c** clinical chemistry and hematology variables (*n* = 30) based on 94 subjects; **d** immune cell profiles from PBMC (*n* = 53) based on 93 subjects; **e** metabolites in plasma (*n* = 413) based on 94 subjects; **f** lipids in plasma (*n* = 169) based on 48 subjects; **g** fecal microbiota based on 16S sequencing and using 1465 operational taxonomic units (OTUs) for 89 subjects; and **h** PBMC transcriptome expression values from 11,976 genes based on 77 subjects. Each plot shows all individuals with complete data from all four visits for the respective datasets, except for the proteome and clinical chemistry data where six visits were analyzed, colored by sex and with lines connecting the visits for each individual. **i** The average distance between visits for each individual calculated per data type. The autoantibody profiling was excluded from this analysis due to the high stability over time. Euclidean distance was used for all methods except microbiota, which used Bray–Curtis distance, and immune cytome, which used Aitchisons distance. The individuals with the top ten largest average distances are highlighted in different colors and all others are shown in gray. **j** Intra-class correlation (ICC) levels in each variable from each dataset. In **j** data are represented as violin plots where the middle line is the median.

In Fig. 3a–g, the integrated molecular profiles of all individuals across all analyzed visits are visualized using two-dimensional maps generated by the dimension reduction technique Uniform Manifold Approximation and Projection (UMAP)^24^. In the resulting plots, all visits of a particular individual are connected by lines, indicating how similar the profile of an individual is between two visits. The animated versions of the plots are presented in Supplementary Movie 2, where the movement of each individual across time can be dynamically tracked.

For the autoantibody data, each of the individuals display a unique and stable global repertoire of autoantibody reactivity during the study period with very little changes in the signature of IgGs binding the selected panel of self-proteins (Fig. 3a). Similarly, the plasma proteome analysis (Fig. 3b) reveals that each individual has its own unique protein profile and shows a remarkable stability with regards to plasma profiles during the study period. In addition, the results reveal two subclusters composed of men and women, respectively, suggesting a variation of the plasma protein profiles between the two sexes, similar to what we have shown for the clinical variables. The comprehensive summary of 30 clinical chemistry variables (Fig. 3c) shows that many individuals have all visits located close to each other, suggesting that a majority of the global clinical chemistry profiles are unique and stable during the study period, although we see a larger variation overtime than for the autoantibody and proteome datasets.

The results from the immune cell profiling (Fig. 3d) show a stable immune cell composition for some individuals, while others change significantly between two or more visits. The number of individuals with variable immune cell profiles is larger than the number of individuals with variable plasma protein and clinical chemistry profiles, highlighting the reactive nature of immune cells in blood. Similarly, the UMAP results for the metabolome (Fig. 3e), lipidome (Fig. 3f), and microbiota (Fig. 3g) show that many of the analyzed individuals have a unique and stable profile, although some show larger changes between two or more visits, suggesting individual-related variability of the corresponding profiles between these visits. Finally, the transcriptomics profiling is the most variable dataset and shows a significant varying fraction overtime (Fig. 3h). As contrary to the clinical and plasma proteome data, neither the transcriptomic, autoantibody, lipidomics, immune cell or microbiota data show any major differences between men and women in the UMAP plots.

### Individual intervisit distances

To assess the changes over visits, we used the molecular profiles of all individuals to calculate the distances between two visits for each individual and each analysis method, based on Bray–Curtis dissimilarity measure^25^ for gut microbiota data, the Aitchison’s distance^26^ for immune cell data and Euclidean distance^27^ for the remaining datasets. Supplementary Fig. 3 shows the scaled pairwise distances between visits for each of the different datasets, and with the ten most highly varying individuals based on mean values for all methods colored separately. To compare the variability for the individuals across different omics datasets, the average of the distances for all individuals are visualized in Fig. 3i, using the same color code. The results reveal that several of the most variable individuals show increased variability across many of the analyzed data types, suggesting simultaneous changes across multiple molecular profiles. As an example, an individual with a significant increase in CRP levels at the second visit (79.0 mg/L) has the largest variation of all in protein plasma levels as well as in clinical chemistry and transcriptomics and is in the top four for both metabolomics and microbiota profiling.

### Intraindividual variation of molecular profiles

To understand the variation of each individual, we performed an intra-class correlation (ICC) analysis^28^ for all variables individually and extracted the variance related to each subject. The results are shown in Fig. 3j for all variables across the eight datasets sorted by the median value and where an ICC level close to one reflects a more stable individual profile. The results show that the autoantibody dataset has the highest ICC median value and thus most distinct and stable individual profiles, while the microbiota showed the lowest ICC median with a wider dispersion which means a higher intraindividual variability. To further track this intraindividual variation, we used *Z*-scores of each variable (feature) in each individual at the dataset level and across all datasets. We used *Z*-score = 2 as a cutoff to define a feature as varying. The overall results are shown in Supplementary Figs. S4 and S5, in which the individuals are sorted according to the total fraction of their varying features by including all datasets. For example, the abovementioned individual (W0022) with high CRP is ranked as the second most varying individual based on aggregated *Z*-scores for all datasets (Supplementary Fig. 4A). Another highly varying individual (W0008) exhibits a high LDL (4.90 mmol/L) and low HDL (1.30 mmol/L) at the fourth visit which explain the shifts overtime. Furthermore, Supplementary Fig. 4B displays the relative variable proportion of each dataset that is represented in the varying fraction in each individual, as well as a summary of the assessed datasets per individual in Supplementary Fig. 4C. This allows for quantification of the driving variation at the individual level. The data show that the top most variable individuals have at most 12% of varying features while the most stable one shows only 1.3% overall variation, confirming the overall stability of the cohort over the study period. Furthermore, this shows how metabolic changes overtime could be tracked at a global level using deferent biological and clinical datasets as biological lenses.

For five of the datasets, the ten top most stable or the top most varying features based on their interquartile ranges (IQR) are shown in Supplementary Fig. 6. Some notable examples of variable proteins include the growth hormone 2 (GH2)^29^ which shows a large range of expression values and the alpha subunit of the glycoprotein hormones (CGA) which shows the expected higher expression in women than men. In addition, we see that among the most stable metabolites we find gamma (γ)-tocopherol^30^, a form of vitamin E. Evidence of γ-tocopherol effect in preventing endothelial injury, lipid peroxidation, and oxidative stress have been reported^30,31^. Indeed, γ-tocopherol is important in preserving endothelial function by protecting the degradation of tetrahydrobiopterin (BH4), a key cofactor in the synthesis of nitric oxide^32^. Furthermore, γ-tocopherol exhibits superior anti-inflammatory and antioxidant pharmacodynamic properties by inhibiting COX-2 and 5-lipoxygenase pathways^33^.

### Correlation-based integrative analysis

**Fig. 4:**
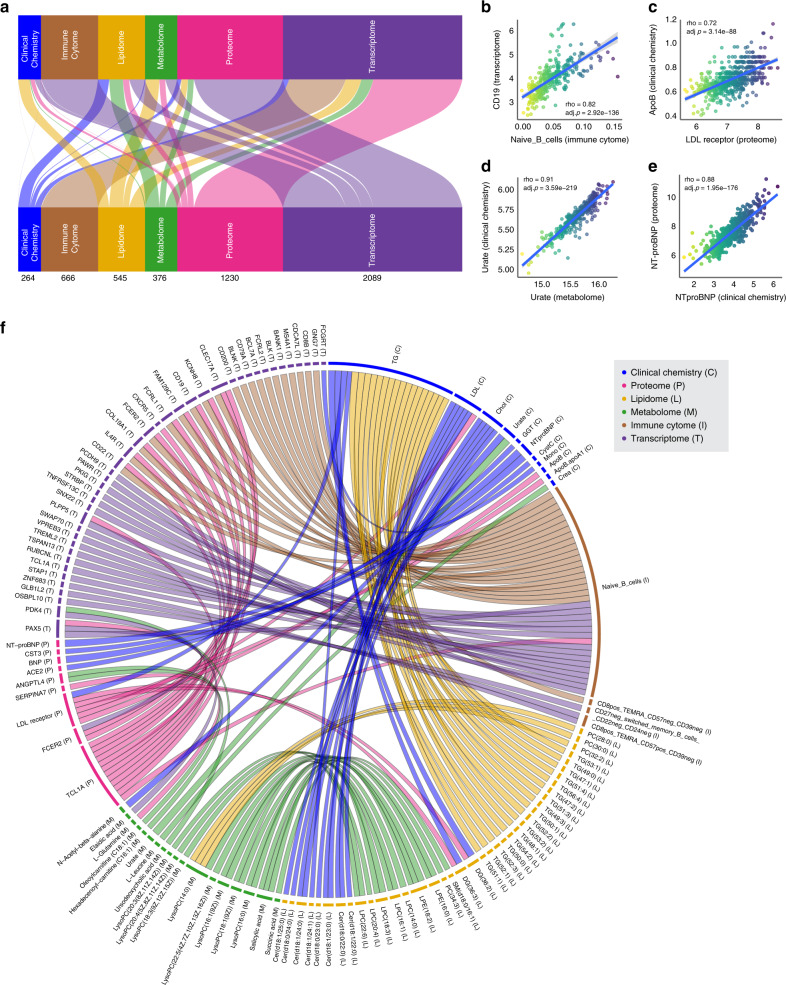
Inter- and intra-omic correlation. **a** Flow diagram showing the combinations of inter-omics Spearman correlations above 0.5 between the different datasets. The numbers represent the number of features correlating between two datasets. Selected examples of highly correlated variables between two datasets using spearman correlation: **b** CD19 and naïve B cells, **c** ApoB and LDL receptor, **d** Urate, and **e** NTproBNP. **f** Chord diagram of the top 20 inter-omic Spearman correlations for each of the datasets. The ribbon thickness reflects the spearman correlation. Multiple test corrections have been applied for *p* values using Benjamini and Hochberg method.

With the comprehensiveness of the assessed omics and clinical data, this study provides an interesting opportunity to identify associations between the different biological and clinical information layers. Thus, we performed intra- and inter-dataset correlations based on Spearman correlation between all analyzed variables included in six of the datasets (*n* = 13,435), excluding the autoantibody and microbiota datasets. Intra-omic correlations is defined as correlations computed between features from the same dataset while inter-omic correlations is defined as correlations between features from different datasets. A complete list of all results above 0.2 or below −0.2 is available at: https://www.proteinatlas.org/download/scapis_wellness_correlation_network_all_data.txt.gz. To explore the inter-omics correlation, we visualized the links between all significant inter-omics correlation above 0.5 or below −0.5 (Fig. 4a). We observed that largest links consist of correlations between the transcriptome with the proteome followed by the transcriptome and the immune cytome, which are both based on analysis of PBMCs. Some of the most highly correlated features include the CD19 molecule, which is highly correlated with the Naive B cells (transcriptomics and immune cytome, *⍴* = 0.82) (Fig. 4b), and the LDL receptor that is highly correlated with Apolipoprotein B (ApoB)^34^ (proteomics and clinical chemistry, *⍴* = 0.72) (Fig. 4c). As expected, we observed high correlation between analytes that are assessed with different technologies such as urate (clinical chemistry and metabolomics, *⍴* = 0.91) (Fig. 4d) and N-terminal pro b-type natriuretic peptide (NTproBNP) (clinical chemistry and proteomics, *⍴* = 0.88) (Fig. 4e), which reflect the consistence of the data across different platforms. A larger selection of examples is shown in Supplementary Fig. 7 and the detailed top 20 pairwise correlations for each of the datasets are visualized in Fig. 4f to gain more insights regarding the most highly correlated features. We find that most of the top immune cytome connections are due to Naive B cells and that the lipidome is mainly correlated with clinical data through the lipid profile biomarkers (triglycerides (TG), cholesterol, and LDL). Most of the proteome correlations are due to T-cell leukemia/lymphoma 1A (TCL1A) which is highly correlated with multiple genes in the transcriptomics data, for example the Fc receptor like 1 (FCRL1) and the Fc fragment of IgE receptor II (FCER2) genes. The metabolome is as expected mainly correlated with the lipidome but also connects to the clinical chemistry through the kidney function biomarker creatinine and urate. We also observed a negative correlation between LILRB2 (Transcriptome) and LILRB2 (Proteome) with *⍴* = −0.70 and it is not clear whether it is a technical artifact or a biologically relevant finding.

### Assessment of the effect of omics datasets on clinical data

**Fig. 5:**
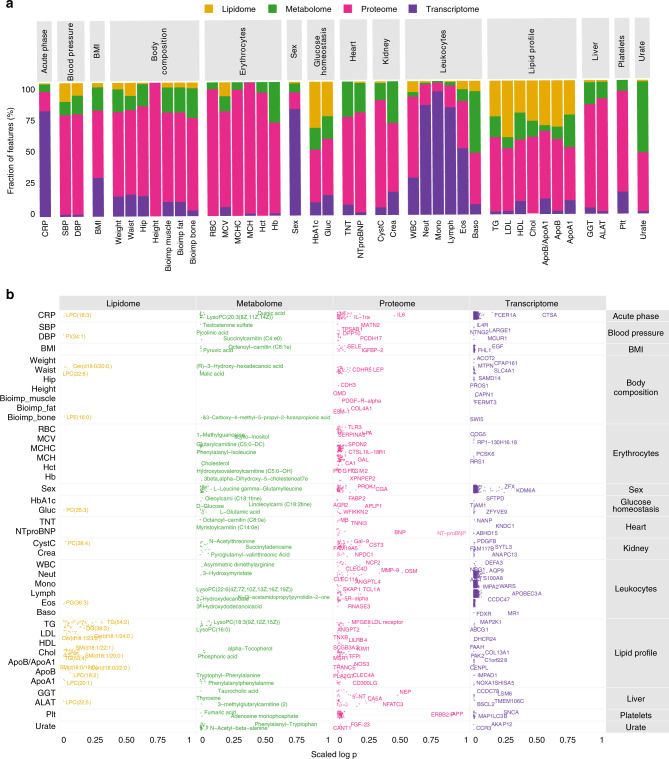
Mixed-effect modeling of four omics datasets versus clinical parameters. **a** Bar plot of the distribution of the most significant results from mixed-effect modeling for each of the clinical variables compared to features from the proteome, transcriptome, metabolome and lipidome. **b** Visualization of the top two most significant features for each clinical data across omics colored by omics type. Multiple test corrections have been applied for *p* values using Benjamini and Hochberg method. Number of samples: metabolome (*n* = 94), lipidome (*n* = 48), proteome (*n* = 90), transcriptome (*n* = 77).

To get a more comprehensive overview of the interrelationships between the omics profiles, anthropometrics, and the clinical chemistry parameters, we performed linear mixed-effect modeling^35^, which creates models for each omic feature and all clinical data while at the same time can take into consideration the effect of sex and individual. The aim of this analysis is to quantify and predict the effect of the omics data on the assessed clinical parameters. Four of the datasets showed significant effects on the clinical data: proteome, lipidome, metabolome and transcriptome, and the complete table with all significant results is given in Supplementary Dataset 3. The overall results (Fig. 5a) reveal that the proteome exhibits most of the effects on the clinical data. The strong connection between blood cells and the PBMC transcriptome is also shown here by the large fraction of transcriptome results in the leukocyte part of the clinical data. Moreover, the data also confirms the relationship between the assessed lipidome and the lipid profile as already mentioned. To have a deeper insight, we present a summary of the two most significant features across the four omics datasets and for each clinical parameter (Fig. 5b) as well as a more detailed view for each dataset in Supplementary Fig. 8. As expected, the lipidome is mainly connected to the lipid profile through TG, sphingomyelines, lysophosphatidylcholines and ceramides. The metabolome had a broader spectrum, such as the alpha tocopherol (vitamin E) that exhibits the highest effect on lipid profile, taurocholic acid is significantly connected to liver biomarker Gamma-glutamyl transferase (GGT). Acylcarnitines presented the highest effect on heart biomarkers Troponin T (TNT) and NTproBNP, which is relevant for such a highly energy consuming organ. Indeed, carnitine and acylcarnitines are important transporters of fatty acids into mitochondria for beta-oxidation which is main source of energy in the heart^36^. Regarding the proteome, the liver function markers alanine aminotransferase (ALAT) showed strong connections with other liver enzymatic proteins Carbonic anhydrase 5A (CA5A) and Hydroxyacid oxidase 1 (HAO1), but also with Angiotensin I converting enzyme 2 (ACE2) predominately expressed in endothelial cells. While CA5A and HAO1 are intracellular liver enzymes whose presence in plasma indicate tissue leakage/liver cell damage with no functional role in plasma, the connection with ACE2, which is expressed as a transmembrane protein with an enzymatically active ectodomain (sACE2) circulating in plasma, may be a functional consequence in response to liver injury. ACE2 is normally not expressed in liver, but has been shown to be upregulated in liver injury^37^ and in experimental models to have protective effects against liver fibrosis^38^. Interestingly, in liver disease/liver fibrosis, the Ang-(1–7) peptide generated by ACE2, is opposing the harmful effects of angiotensin II on liver and rat models showed it to be protective against liver fibrosis^39^. Our data thus suggest that in normal healthy individuals, there could be a counter regulatory effect in response to mild/subclinical liver injury. We also analyzed the mixed-effect modeling results with regards to the sex-associated variables and the most significant findings per dataset is presented in Supplementary Fig. 9.

### Tissue specificity of protein variation

**Fig. 6:**
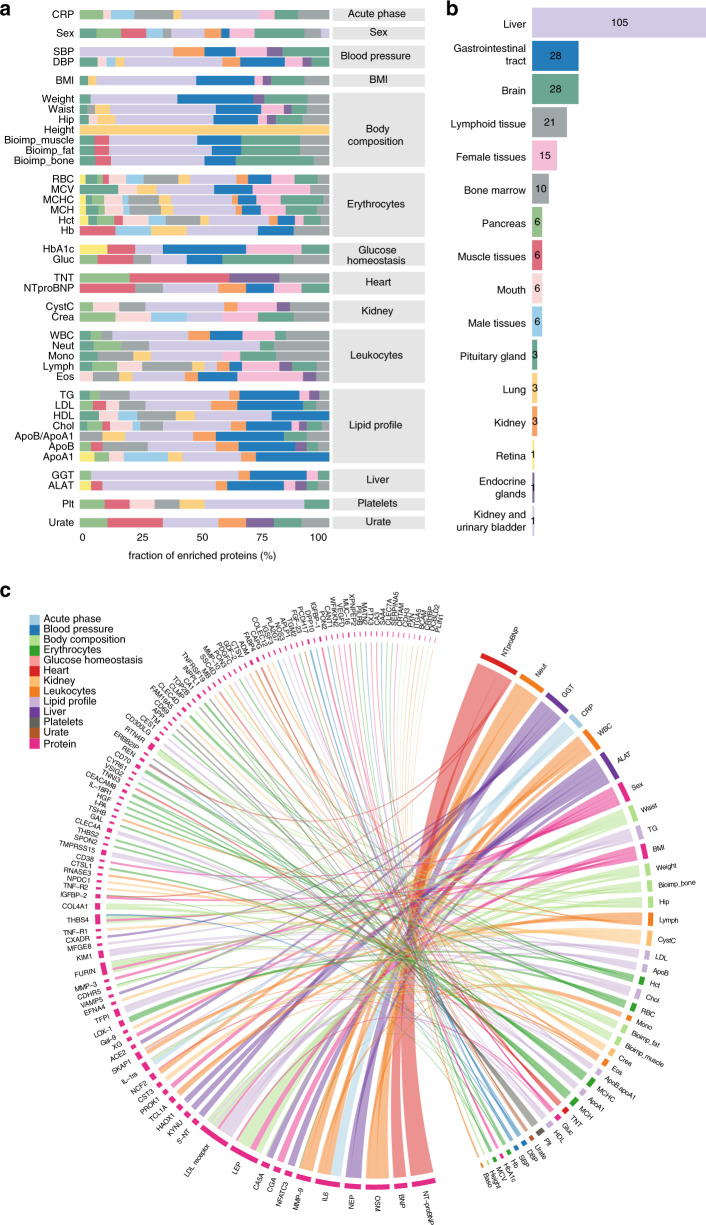
Tissue enrichment analysis of the blood proteome retrieved from mixed-effect modeling. Distribution of tissue enriched proteins in (**a**) each of the clinical parameters classified based on the tissue type and protein profiling results and using the color code in (**b**), with a bar plot showing the distribution of the number of tissue enriched proteins for each tissue type. **c** Chord diagram of the top five most associated proteins for each of the clinical parameters, colored by clinical variable class. The ribbon thickness is proportional to the -log adjusted p-value.

Subsequently, given the metabolic coverage of the assessed blood proteome, we investigated the tissue specificity of the proteins associated with the various clinical parameters. This tissue enrichment step is based on the classification scheme described in Uhlén et al.^20,40^, which classified ~2845 genes as tissue enriched, defined as at least fourfold higher transcript expression levels in one tissue type as compared with any other analyzed tissues, in a total of 37 different cells, tissues, and organs. The list of tissue enriched genes was mapped to the list of plasma proteins with significant effects on any clinical parameter and Fig. 6a shows the distribution of tissue enriched genes from different tissue types across all clinical parameters. Furthermore, Fig. 6b shows the number of tissue enriched genes across all clinical parameters, whereas Fig. 6c visualizes the top five most associated proteins by each of the clinical parameters. The analysis shows that many of the plasma proteins that are highly associated with the clinical data are also expressed by the liver. The proteins encoded by gastrointestinal tract enriched genes are mainly associated with lipid profile, HbA1c (Hemoglobin A1c), BMI, and weight, while bone marrow and lymphoid enriched genes showed high correlation to leukocytes, RBC and related metrics.

At a functional level, we performed an enrichment analysis based on the mixed-effect modeling results and KEGG pathways^41^ and identified 69 significantly enriched metabolic pathways for at least one clinical parameter (Benjamini–Hochberg-adjusted *p* < 0.05) (Supplementary Fig. 10). For example, the result shows that proteins associated with CRP, white blood cell (WBC) count, neutrophil particles (Neut) and Cystatin C (CystC) are involved in the immune system, while some well-known obesity-related pathways like PI3K/Akt, JAK/STAT, RAP1, and MAPK signaling pathways were identified in weight, BMI, waist, and fat associated proteins^42^. Among them, one interesting significant association was for weight, waist, CRP, and WBC involved in Relaxin signaling pathway, which has been reported to have a function in the treatment of diet-induced insulin resistance and vascular dysfunction in mice model^43^.

## Discussion

Several projects aiming to perform molecular profiling of various cohorts have been previously reported with different scopes, sizes and depths^4–8^. The primary objective of this study has been to analyze the global molecular omics profiles of one hundred individuals and to investigate the longitudinal stability and interconnections of such profiles, aiming for a deep, and comprehensive profiling. The analyses are based on molecular profiles in blood complemented with gut microbiota profiles to allow multi-omics data to be integrated and compared with clinical chemistry and other metadata. The individuals were selected to be clinically healthy, but all individuals enrolled to the study started with an in-depth screening using an extended medical imaging and a battery of clinical chemistry assays. Thus, many of them were found to have either minor or major disorders, emphasizing the difficulties to define the healthy state of individuals. We show that each individual has a unique protein profile and autoantibody profile and that in most cases these profiles are stable overtime in the absence of infectious disease or any other acute perturbation. It is also noteworthy that the other omics profiles across the visits for a particular individual also, in many cases, cluster together and are typically distinct from the profiles of other individuals.

This study is, as far as we know, one of the most comprehensive and deep longitudinal analyses of omics yet published and this was achieved using different multimodal technologies. Broadly, the gathered proteomic data offer a tremendous wealth of information to empower routine laboratory tests with a more biologically contextualized and clinically relevant insight for a better data interpretation in both translational and clinical settings. The study shows that the longitudinal shifts between individuals are higher than the intraindividual variation in all datasets. This highlights the need for a higher biological granularity in tracking variation at a more individual scale^44^.

The longitudinal plasma protein profiles show that a large majority of the proteins were stable in the individuals and only a few showed significant differences between the individual participants. This agrees with the finding that signatures of circulating proteomes are highly individual specific^45^. In addition, we recently reported that the human blood level of many proteins during adult life is to a large extent affected by genetics, confirming the unique and stable individual patterns of the proteome profiles^46^. Among the most variable proteins we find human growth hormone 1 and 2 (GH1 and GH2)^29^, which may be depending on the large variation in expression levels between men and women with growth hormone levels reported to be sex, age, BMI and exercise dependent^47^. An interesting finding is the sex-associated differences underlined by complex metabolic and endocrine interconnections, which is important for our understanding of both health and disease to avoid sex biased interpretations. These differences are clearly reflected in the variation in clinical laboratory parameters between females and males, and sex is a key factor in establishing reference intervals^48^, such as endocrine specificities and skeletal muscle mass metabolism^49,50^. Interestingly, our data show sex-related differences for many of the parameters related to metabolic syndrome risk factors^51^. Moreover, lipoprotein profiles are highly sex-related given the greater insulin sensitivity of women compared to men^52^. Thus, our data emphasize the importance of accounting for metabolism differences between the sexes as a key individual attribute to effectively implement personalized, preventive, and therapeutic strategies.

The analysis of the individual molecular profiles revealed large effects on several parameters. The liver marker GGT was identified in relation to inflammatory markers such as IL-6 and this is not unexpected since the liver plays a pivotal role in inflammation by draining circulating lipids (TG and free fatty acids), and triggering cytokines release, which in turn promote CRP hepatocyte production^53^. The strong relationship between CRP and IL-6 has been recently reported along with other cytokines^46^, and the integrated CRP-centric perspective may shed light on CRP roles in inflammatory-based disorders. This study also involves a very comprehensive longitudinal profiling of immune cell composition in healthy individuals^54^. Here we show that although some individuals are stable overtime, many subjects show a variable immune cell composition, even without overt infectious disease.

Beyond the insightful molecular profiling depth of the current study, the reported observations need to be validated in larger cohorts given the relatively small size of this study cohort.

In summary, we present a deep phenotyping study, aiming to contribute to laying a foundation and reference for future precision medicine endeavors based on molecular monitoring of wellness and deviations from an individual trajectory perspective as an early sign of disease development. The results support an individual-based definition of health and show a path forward for precision medicine based on comprehensive molecular profiling in a longitudinal manner.

## Methods

### Study subjects

A total of 101 subjects were recruited from the ongoing Swedish CArdioPulmonary bioImage Study (SCAPIS), which is a prospective observational study of ~30,000 subjects aged 50–64 years from the general Swedish population, enrolled to the study based on tax registry personal number to allow a random sampling of the population and subsequent follow-up via national registries. Examinations in SCAPIS include imaging to assess coronary and carotid atherosclerosis, clinical chemistry, anthropometry, and extensive questionnaires, as previously described^15^. Thus, the subjects had been extensively phenotyped before entering the S3WP program. In SCAPIS, no exclusion criteria are applied except the inability to understand written and spoken Swedish for informed consent. In the S3WP program, exclusion criteria include: (1) previously received health care for myocardial infarction, stroke, peripheral artery disease or diabetes, (2) the presence of any clinically significant disease which, in the opinion of the investigator, may interfere with the results or the subject´s ability to participate in the study, (3) any major surgical procedure or trauma within 4 weeks of the first study visit, or (4) medication for hypertension or hyperlipidemia. The study is approved by the Ethical Review Board of Göteborg, Sweden. All participants provided written informed consent. The study protocol conforms to the ethical guidelines of the 1975 Declaration of Helsinki.

### Study design

The S3WP program is non-interventional with the aim to collect longitudinal data in a community-based cohort. A total of four examinations were performed every third month (±2 weeks) for the first round and an additional two examinations were performed approximately every 6 months for the second round. The individuals in this study are part of the larger SCAPIS study and were identified as clinically healthy based on their results. One hundred one individuals were recruited to the program, 99 completed the first round and 94 completed the second round. Clinically healthy was defined as previously not received health care for myocardial infarction, stroke, peripheral artery disease or diabetes or having other clinically significant disease, not having had major surgical procedures or trauma within four weeks of the first study visit and not taking medication for hypertension or hyperlipidemia. All subjects were fasting overnight (at least 8 h) before the visits. Subjects underwent the same examinations at each visit, including anthropometric measurements, body fat using bioimpedance and blood pressure. A selection of questions from the initial SCAPIS questionnaire was repeated to note any changes in health and lifestyle factors between each visit. Each visit also included collection of blood, urine and feces for subsequent clinical chemistry and omics analyses. From visit two and onward, subjects were wearing accelerometers (Polar A360) to measure physical activity.

Characteristics based on the SCAPIS data are shown in Supplementary Table 1. Self-reported health issues during the study period involved mostly mild infections, with a total of 44 subjects reporting symptoms of a common cold and 13 subjects reporting bacterial infections. Fourteen subjects underwent some form of surgery, ten subjects started antihypertensive medication, and five subjects started statin treatment during the study period. The mean body weight remained stable during the observation period, with the exception of one subject experiencing marked diet-induced weight loss, −15.8 kg (−34.8 pounds) between visits 3 and 4, as detailed below. The clinical chemistry variables were largely stationary at the group level and the most noticeable finding at the individual level was a pronounced CRP elevation (79.0 mg/L) at visit 2 in one subject.

### Clinical examinations

Height was measured in indoor clothing to the nearest centimeter without shoes. Weight was measured on a calibrated digital scale, with subjects dressed in light indoor clothing without shoes. The BMI was calculated by dividing the weight (kg) by the square of the height (m). Waist circumference was measured midway between the palpated iliac crest and the palpated lowest rib margin in the left and right mid-axillary lines. Hip circumference was measured at the maximum circumference over the buttocks. Bioimpedance was measured using Tanita MC-780MA according to manufacturer’s instructions. Systolic and diastolic pressure was registered in supine position and after 5 min of rest, using the automatic Omron P10. The blood pressure was measured in both arms at visit 1 and thereafter in the arm that showed the highest blood pressure at visit 1.

### Clinical chemistry

Clinical chemistry and hematology measurements included capillary glucose (Hemocue), plasma glucose, HbA1c, TG, total cholesterol, LDL, HDL, ApoA1, ApoB, ApoA1/B ratio, creatinine, high sensitive C-reactive protein (hsCRP), ALAT, GGT, urate, cystatin C, vitamin D, TNT, NTproBNP, Hb and a complete blood count including differential.

### Questionnaires

A questionnaire, administered already in the SCAPIS trial, comprising 140 questions separated in sets relating to factors central to the research aims, was used to collect detailed information on self-reported health, family history, medication, occupational and environmental exposure, lifestyle, psychosocial well-being, socioeconomic status and other social determinants. A food-frequency questionnaire (Mini-Meal-Q) with 35 questions was also used. At each visit in the S3WP program, a selection of questions was repeated that will update the information of the initial SCAPIS questionnaire. Subjects were also asked about changes in lifestyle factors between each visit such as infections, disease, medication, perceived health, and exercise level. The Mini-Meal questionnaire was repeated at all visits.

### Metabolomics

Metabolites were extracted from plasma after protein precipitation with methanol^55^. The metabolite extracts were analyzed by Agilent 1290 Infinity UHPLC-system (reverse phase chromatography) combined with an Agilent 6550 Q-TOF mass spectrometer equipped with an electrospray Jetstream ion source operating in positive and negative ion mode. The *m*/*z* range was 70–1700, and data were collected in centroid mode with an acquisition rate of 4 scans/s. The mass spectrometry files were processed using Profinder B.08.01 (Agilent Technologies Inc., Santa Clara, CA, USA) using mass feature extraction for peak detection.

### Lipidomics

The lipid content were extracted following a modified Folch protocol^56–58^. In detail, 250 µL of extraction buffer (2:1 v/v chloroform:methanol) including internal standards (tripalmitin TG(16:0/16:0/16:0)-1,1,1-^13^C3, Ceramide(d18:1/16:0-d31), phosphatidylcholine PC(18:0/18:0-d70) and distearin DG(18:0/0:0/18:0)-d5) were added to 20 µl of plasma and 30 µl of 0.15 M NaCl. The sample was shaken at 30 Hz for 2 min in a mixer mill, and then proteins were precipitated at room temperature for 1 h. The sample was centrifuged at +4 °C, 14,000 rpm, for 3 min. 120 µL of the lower phase were collected and divided into two different microvials (40 + 80 µL) and stored at −80 °C until LC/MS analysis. In total, 200 µL of extraction buffer (2:1 v/v chloroform:methanol) including internal standards (tripalmitin-1,1,1-13C3 and 16:0-d31 ceramide) were added to 150 pancreatic islets. The sample was shaken with a tungsten bead at 30 Hz for 2 min in a mixer mill, the beads were removed and 40 µl of 0.1 M NaCl was added. After vortex for 2 min, the samples were let to stand at room temperature for 30 min. The sample was centrifuged at +4 °C, 14 000 rpm, for 3 min. 120 µL of the lower phase were collected and divided into two different microvials (40 + 80 µL) and stored at −80 °C until analysis. The LC–MS analysis of the lipid extracts were performed as described^56^. The chromatographic separation was performed on an Agilent 1290 Infinity UHPLC-system (Agilent Technologies, Waldbronn, Germany). 0.5 µL of extracted plasma or 1 µl of extracted tissue sample were injected onto a Acquity UPLC CSH, 2.1 × 50 mm, 1.7 µm C18 column in combination with a 2.1 mm × 5 mm, 1.7 µm VanGuard precolumn (Waters Corporation, Milford, MA, USA) held at 60 °C. The gradient elution buffers were A (60:40 acetonitrile:water, 10 mM ammonium formate, 0.1% formic acid) and B (89.1:10.5:0.4 2-propanol:acetonitrile:water, 10 mM ammonium formate, 0.1% formic acid), and the flow rate was 0.5 mL min^−1^. The compounds were eluted with a linear gradient using initial condition 15% B, and increase to 30% B at 1.2 min, 55% at 1.5 min, isocratic to 5.0 min, increase to 72% B at 7 min, 85% at 9.5 min and 100% B at 10.0 min, and then held at 100% for 2 min. An additional wash of the injection valve, with 100% B and flow rate 3.0 mL min^−1^ for 0.3 min, was performed before decreased to initial condition 15% B over 0.3 min; these conditions were held for 1.1 min to equilibrate the column before next injection. The compounds were detected with an Agilent 6540 Q-TOF mass spectrometer equipped with a dual jetstream electrospray ion source operating in positive or negative ion mode. The settings were kept identical between the modes, with exception of the capillary voltage. A reference interface was connected for accurate mass measurements; the reference ions purine (2 µM) and HP-0921 (Hexakis(1H, 1H, 3H-tetrafluoropropoxy)phosphazine) (2.5 µM) both purchased from Agilent Technologies (Santa Clara, CA, USA) were infused directly into the MS at a flow rate of 0.07 mL min^−1^ for internal calibration, and the monitored ions were purine *m*/*z* 121.05087 and *m*/*z* 119.03632; HP-0921 m/z 922.00980 and *m*/*z* 966.000725 for positive and negative mode respectively. The gas temperature was set to 300 °C, the drying gas flow to 8 L min^−1^ and the nebulizer pressure 40 psig. The sheath gas temp was set to 350 °C and the sheath gas flow 11 L min^−1^. The capillary voltage was set to 4000 V in positive ion mode, and to 4000 V in negative ion mode. The nozzle voltage was 0 V. The fragmentor voltage was 100 V, the skimmer 45 V and the OCT 1 RF Vpp 750 V. The collision energy was set to 0 V. The m/z range was 70–1700, and data were collected in centroid mode with an acquisition rate of 4 scans s^−1^ (1973 transients/spectrum). The data were processed using Batch Targeted Feature Extraction algorithm within MassHunter™ ProFinder version B.08.00 (Agilent Technologies Inc., Santa Clara, CA, USA). In-house database with exact mass and experimental retention times of lipids were used for identification.

### Plasma protein profiling

Plasma proteins were analyzed using a multiplex proximity extension assay (Olink Bioscience, Uppsala Sweden). Each kit provides a microtiter plate for measuring 92 protein biomarkers in 90 samples. Each well contains 96 pairs of DNA-labeled antibody probes. Samples were incubated in the presence of proximity antibody pairs. In short, 1 µL sample (buffer (PBS with 0.1% BSA), antigen-spiked buffer, or biological sample) was mixed with 0.3 µL of each proximity probe mix (A and B), 0.3 µL Incubation Stabilizer (Olink Proteomics, Uppsala, Sweden) and 2.1 µL Incubation Solution (Olink Proteomics) and incubated overnight at 4 °C. A combined extension and preamplification mix (96 µL) containing 10 µL MUX PEA Solution (Olink Proteomics), 0.5 units Pwo (DNA Gdansk, Poland), 1 µM forward + reverse universal preamplification primers, and 1 unit hot-start DNA polymerase was added to each reaction at RT. After mixing and a total 5-min incubation, the plate was transferred to a thermocycler running an initial extension step to unite the two oligonucleotides (50 °C, 20 min), immediately followed by a hot-start step (95 °C, 5 min) and 17 cycles of amplification (95 °C, 30 s; 54 °C, 1 min; 60 °C, 1 min). Amplification was performed with universal flanking primers to amplify all 96 sequences in parallel. Finally, 2.8 µL of the preamplification products were mixed with 7.2 µL buffer containing 5 µL MUX Detection Solution (Olink Proteomics), 0.071 units Uracil-DNA glycosylase (DNA Gdansk) used to digest the DNA templates and remaining universal primers, and 0.14 units hot-start polymerase. In total, 5 µL from the sample mix above was transferred to the sample inlet wells of a microfluidic real-time PCR chip (96.96 Dynamic Array IFC, Fluidigm Biomark). In total, 5 µL from respective well of an Assay Plate (Olink Proteomics) containing 9 µM sequence-specific internal detection primers, 2.5 µM molecular beacon in 1× DA Assay Loading Reagent (Fluidigm) were transferred to the assay inlet wells). The chip was run in a Biomark instrument with the following program: Thermal mix (50 °C, 2 min; 70 °C, 30 min; 25 °C; 10 min), Hot-start (95 °C, 5 min), PCR Cycle 40 cycles (95 °C, 15 s; 60 °C, 1 min) according to the manufacturer’s guidelines (http://www.fluidigm.com/biomark-hd-system.html).

To minimize inter- and intra-run variation, the data are normalized using both an internal control (extension control) and an interplate control, and then transformed using a predetermined correction factor. The pre-processed data were provided in the arbitrary unit Normalized Protein eXpression (NPX) on a log2 scale and a high NPX presents high protein concentration. In this study, 11 Olink panels have been used including Cardiometabolic, Cell Regulation, Cardiovascular II (CVD II), Cardiovascular III (CVD III), Development, Immune Response, Immuno-Oncology, Oncology II, Inflammation, Metabolism, Neurology, and Organ Damage. Since samples were analyzed at two different locations and at different time points, two strategies were used to assess the batch effect of sampling: (1) the ratio of maximum and minimum IQR of protein expressions across six visits; (2) three-way ANOVA analysis^59^ of protein expressions for factor batch number, factor visit and factor subject. Proteins with the ratio of maximum and minimum IQR > 1.8 or coefficient of batch from ANOVA > 10 were considered to have a problematic batch effect and were removed from the dataset. Thirty-nine replicated proteins from multiple panels were also removed. The filtering process resulted in a total of 794 unique proteins that were used in the analysis of this study.

### Transcriptomics profiling

Total RNA was extracted using RNeasy Mini Kit (Qiagen) and quantified using Qubit 2.0 Fluorometer (Invitrogen). RNA was converted to a sequencing library using the TruSeq Stranded mRNA HT library preparation method (Illumina) using 500 ng of total RNA as input quantity. The obtained library was quantified using Qubit Broad Range assay kit or Quant-IT RiboGreen chemistry (Invitrogen). The obtained libraries were sequenced using Hiseq 2500 (Illumina) using either pair-end 100 bp or pair-end 125 bp in rapid run mode or high output mode, respectively. Each sample was sequenced targeting 30 M read pairs. Demultiplexing was done without allowing any mismatches in the index sequences. To obtain quantification scores for all human genes and transcripts across all samples, transcript expression levels were calculated as transcript per million (TPM) by mapping processed reads to the human reference genome GRCh37/hg19 and with gene models based on Ensembl (v92)^60^ using Kallisto (v.0.43.1)^61^. Data from multiple visits were integrated using batch correction implemented as removeBatchEffect in the R package Limma^62^ using the sampling date as a batch parameter. This resulted in gene expression data for 19,670 genes out of which 11,976 were detected in the 77 analyzed individuals based on a cutoff for detection of average TPM > 1 per gene.

### 16S rRNA gene profiling of human fecal microbiota

The fecal microbiota was profiled by sequencing the V4 region of the 16S rRNA gene on an Illumina MiSeq instrument (Illumina RTA v1.17.28; MCS v2.5) with 515F and 806R primers designed for dual indexing^23^ and the V2 Illumina kit (2 × 250 bp paired-end reads). 16S rRNA genes were amplified in duplicate reactions in volumes of 25 μL containing 1× Five Prime Hot Master Mix (5 PRIME GmbH), 200 nM of each primer, 0.4 mg/ml BSA, 5% DMSO, and 20 ng of total fecal genomic DNA. PCR was carried out under the following conditions: initial denaturation for 3 min at 94 °C, followed by 25 cycles of denaturation for 45 s at 94 °C, annealing for 60 s at 52 °C, and elongation for 90 s at 72 °C, and a final elongation step for 10 min at 72 °C. Duplicates were combined, purified with the NucleoSpin Gel and PCR Clean-up kit (Macherey-Nagel), and quantified using the Quant-iT PicoGreen dsDNA kit (Invitrogen). Purified PCR products were diluted to 10 ng/μL and pooled in equal amounts. The pooled amplicons were purified again using Ampure magnetic purification beads (Agencourt) to remove short amplification products. Amplicons were sequenced on a Illumina MiSeq, 150 cycles paied-end using SBS reagents v2. For MiSeq.

Illumina reads were merged using PEAR and quality filtered by removing all reads that had at least one base with a q-score lower than 20^63^. Quality filtered reads were analyzed with the software package QIIME (version 1.9.1)^64^. Sequences were clustered into OTUs at a 97% identity threshold using an open-reference OTU picking approach with UCLUST^65^ against the Greengenes reference database, 13_8 release^66^. Representative sequences for the OTUs were Greengenes reference was taxonomically assigned using the Greengenes taxonomy and the Ribosomal Database Project Classifier^67^. Representative OTUs were aligned using PyNAST^68^ and used to build a phylogenetic tree with FastTree^69^, which was used to calculate α- and β-diversity of samples using Phylogenetic Diversity^70^ and UniFrac^71^. Chimeric sequences were identified with ChimeraSlayer^72^ and excluded from all downstream analyses. Similarly, OTUs that could not be aligned with PyNAST, singletons and low abundant OTUs with a relative abundance < 0.002% were also excluded. We obtained an average of 61,997 ± 17,134 sequences/sample (mean ± SD; range 16,663–144,133 sequences/sample); a total of 23,310,906 sequences and 1465 OTUs were included in the analyses. A rarified counts matrix has been used for this study.

### Autoantibody profiling

Autoantibody profiling was conducted by measuring subject IgG reactivity toward a selected set of 335 protein fragments produced within the Human Protein Atlas project^19,20^. In short, the protein fragments were coupled to color coded magnetic spheres (MagPlex, Luminex Corp), and the presence of self-reactive IgG in the subjects’ sera was detected by a fluorescent anti-human IgG. Fluorescent data were transformed in relation to sample specific baselines into 17 different reactivity scores, which were used to map similarities between individuals as well as distances between the four visits.

### Cell analyses by mass cytometry

Cryopreserved PBMCs obtained by ficoll density gradient centrifugation from heparinised blood samples of Swedish individuals aged 50–65 (were thawed using RPMI medium (HyClone^®^) supplemented with fetal bovine serum (FBS), penicillin-streptomycin and benzonase (Sigma-Aldrich, St. Louis, MO, USA) and rested overnight at 37 °C in 5% CO_2_ for cells to be revitalized. Cells were then counted and checked for their viability. For live-dead discrimination, cells were stained with 2.5 μM Cisplatin (Fluidigm, South San Francisco, CA, USA) in RPMI without FBS for 5 min at room temperature, followed by quenching with RPMI containing FBS. Cells were then resuspended in CyFACS buffer (PBS with 0.1% BSA, 0.05% sodium azide and 2 mM EDTA) and transferred to a 96-well ‘U’ bottom plate. For surface marker staining using automation (Agilent Technologies, Santa Clara, CA, USA), cells were incubated for 30 min at 4 °C with a 30ul cocktail of metal conjugated antibodies targeting the surface antigens, washed with CyFACS buffer and fixed with 4% formaldehyde overnight, following which cells were stained with DNA intercalator (0.125 μM MaxPar^®^ Intercalator-Ir, Fluidigm Inc.). Cells were subsequently washed with CyFACS buffer, PBS and milliQ water, filtered through a 35 µm nylon mesh, diluted to 500,000 cells/ml and acquired at a rate of 300–500 cells/s using a CyTOF2 (Fluidigm) mass cytometer, CyTOF software version 6.0.626 with noise reduction, a lower convolution threshold of 200, event length limits of 10–150 pushes, a sigma value of 3, and flow rate of 0.045 mL/min. A total of 78,891,056 cells were analyzed across all samples and known marker combinations were used to define 115 canonical immune cell populations across all immune cell lineages and covering multiple activation and differentiation states within these lineages. The 53 most widely abundant and robustly detected cell populations were included in the further analyses.

### Mass cytometry antibodies and reagents

Purified antibodies were obtained in carrier/protein-free buffer and then coupled to lanthanide metals using the MaxPar X8 antibody conjugation kit (Fluidigm Inc.) as per the manufacturer’s recommendations. Metal conjugated antibodies were also purchased from Fluidigm. The antibodies used for this study are listed in Lakshmikanth et al.^54^.

### Statistical analysis

Before multivariate modeling, data were log transformed and scaled. For microbiota data, we used centered log ratio as a log transformation method that addresses compositionality in microbial data^73^. PCA has been applied as an unsupervised multivariate modeling method for dimension reduction and to get an overview of the clustering trends of samples with similar data profiles. Most data analysis was performed using the R project for statistical computation^74^. Mixed-effect modeling was performed using the lme4 package^75^ and Kenward–Roger approximation^76^ was used to calculate *p* values which were subsequently adjusted for multiple testing based on false discovery rate using Benjamini and Hochberg method. *p* values were considered significant if <0.05. For transcriptomic data, X and Y chromosome genes have been removed from the analysis. To evaluate the dispersion pattern of values per analyte across the whole cohort and within each subject, we used IQR to describe this variation. Analytes were first standardized with a standard deviation of 1 centered at 0 before applying IQR function from the R stats package. To account baseline variability, *Z*-scores were calculated for each analyte after log2-transformation. For *Z*-scores, highly varying features were defined as *Z*-score > 2 for each analyte. The highly varying proportion across datasets was calculated by normalizing the number of features with *Z*-score > 2 from each assay to the total number of analytes profiled with the corresponding assay. The percentage of varying features across assays in each participant was then normalized to 100%. The coefficient of variation, defined as the ratio of the standard deviationto the mean normalized to 100%, was also used to evaluate the dispersion of the feature levels. Ontology and enrichment analysis of mixed-effect modeling results was performed using the Enrichr package^77^. Pathways with adjusted p values (Benjamini and Hochberg method) of <0.05 were considered to be significant. UMAP analysis was performed using the umap package^24^. Euclidean distances between visits were calculated using the base R function dist. Aitchison distances were calculated using the R package robCompositions. Bray–Curtis distances were calculated using the R package. ICC was used as the proportion of total variation explained by subject in the cohort to assess the stability overtime. The ICC R package^28^ has been used to compute the ICC. The correlation analysis was performed using the cor function in R, after sex correction using the sva R package^78^. Correlations with adjusted *p* values (Benjamini and Hochberg method) of <0.05 were considered to be significant.

### Reporting summary

Further information on research design is available in the Nature Research Reporting Summary linked to this article.

## Supplementary information

Supplementary Information

Supplementary Data 1

Supplementary Data 2

Supplementary Data 3

Supplementary Movie 1

Supplementary Movie 2

Description of Additional Supplementary Files

Reporting Summary

## Data Availability

Summary statistics data are available in the supplementary material and the full inter-omic correlation network dataset is available at: https://www.proteinatlas.org/download/scapis_wellness_correlation_network_all_data.txt.gz. The participant-level datasets used for this report have been deposited with the Swedish National Data Service (https://snd.gu.se/, a data repository certified by Core Trust Seal) 10.5878/rdys-mz27. Due to patient consent and confidentiality agreements, the datasets can be made available for validation purposes by contacting snd@snd.gu.se. Data access will be evaluated according to Swedish legislation. Data access for research related questions in the S3WP program can be made available by contacting the corresponding author.

## Code availability

The code can be made available upon request from the corresponding author.
