# Supplementary material for: Integration of molecular profiles in a longitudinal wellness profiling cohort
Source: Nat Commun. 2020 Sep 8;11:4487. doi: 10.1038/s41467-020-18148-7 (PMC7479148; doi:10.1038/s41467-020-18148-7)
Supplement: Supplementary file 1 — Supplementary Information [file 41467_2020_18148_MOESM1_ESM.pdf]

Supplementary Materials for

Tebani et al. Integration of molecular profiles in a longitudinal wellness profiling cohort

The PDF file includes:

Supplementary Table 1. Summary of the characteristics for 99 individuals that completed the first four visits.

Supplementary Figure 1. Longitudinal distribution for a selection of clinical parameters.

Supplementary Figure 2. Hierarchical clustering of clinical chemistry and hematology variables.

Supplementary Figure 3. Individual inter-visit distance per dataset.

Supplementary Figure 4. Summary of the individual variation.

Supplementary Figure 5. Individual variability across datasets

Supplementary Figure 6. Top most stable and most varying variables based on interquartile ranges.

Supplementary Figure 7. Examples of highly correlated variables between datasets.

Supplementary Figure 8. Mixed effect results for clinical variables across omics.

Supplementary Figure 9. Top sex-associated variables retrieved from mixed-effect modeling.

Supplementary Figure 10. KEGG pathway enrichment analysis of mixed effect modeling results.

Other Supplementary Material for this manuscript includes the following:

Supplementary Movie 1. Animation of the longitudinal distribution for a selection of clinical parameters.

Supplementary Movie 2. Animation of the two dimensional UMAP results.

Supplementary Dataset 1. Description and summary of the longitudinal clinical data for the 94 subjects that completed the study.

Supplementary Dataset 2. Complete list of analyzed variables per dataset.

Supplementary Dataset 3. Complete list of significant mixed effect modelling result.

Supplemental tables

Supplementary Table 1. Summary of the characteristics for 99 individuals that completed the first four visits.

	Total	Women	Men
**n (men/women)**	**99**	**51**	**48**
**Age (years)**	**56.9 ± 4.2**	**57.1 ± 4.0**	**56.6 ± 4.4**
Place of birth			
**Sweden (n)**	**84**	**41**	**43**
**Other European countries (n)**	**10**	**8**	**2**
**Non-European countries (n)**	**4**	**2**	**2**
Education (highest)			
**Primary school (n)**	**4**	**1**	**3**
**Secondary school n (n)**	**48**	**27**	**21**
**University (n)**	**45**	**22**	**23**
Lifestyle			
**Never smoked (n)**	**54**	**28**	**26**
**Previous smoker (n)**	**40**	**20**	**20**
**Current smoker (n)**	**3**	**3**	**0**
**No alcohol consumption (n)**	**4**	**2**	**2**
**Moderate alcohol consumption (n)**	**90**	**48**	**42**
**High alcohol consumption (n)**	**4**	**1**	**3**
**Physically active time (%) ***	**6.5 ± 2.7**	**6.2 ± 2.5**	**6.7 ± 2.9**
**Sedentary time (%) ***	**56.5 ± 9.0**	**55.5± 8.7**	**57.6 ± 9.2**
Chronic diseases or medication reported by >1 subject			
**Asthma (n)**	**4**	**3**	**1**
**Hypothyreosis (n)**	**3**	**0**	**3**
**Selective serotonin reuptake inhibitors (n)**	**3**	**1**	**2**
**Hormone replacement therapy (n)**	**2**	**0**	**2**

* Physical activity was measured using accelerometer as percent of valid wear time spent in moderate to vigorous physical activity (MVPA), and sedentary time as percent of valid wear time being physically inactive.

Supplemental figures

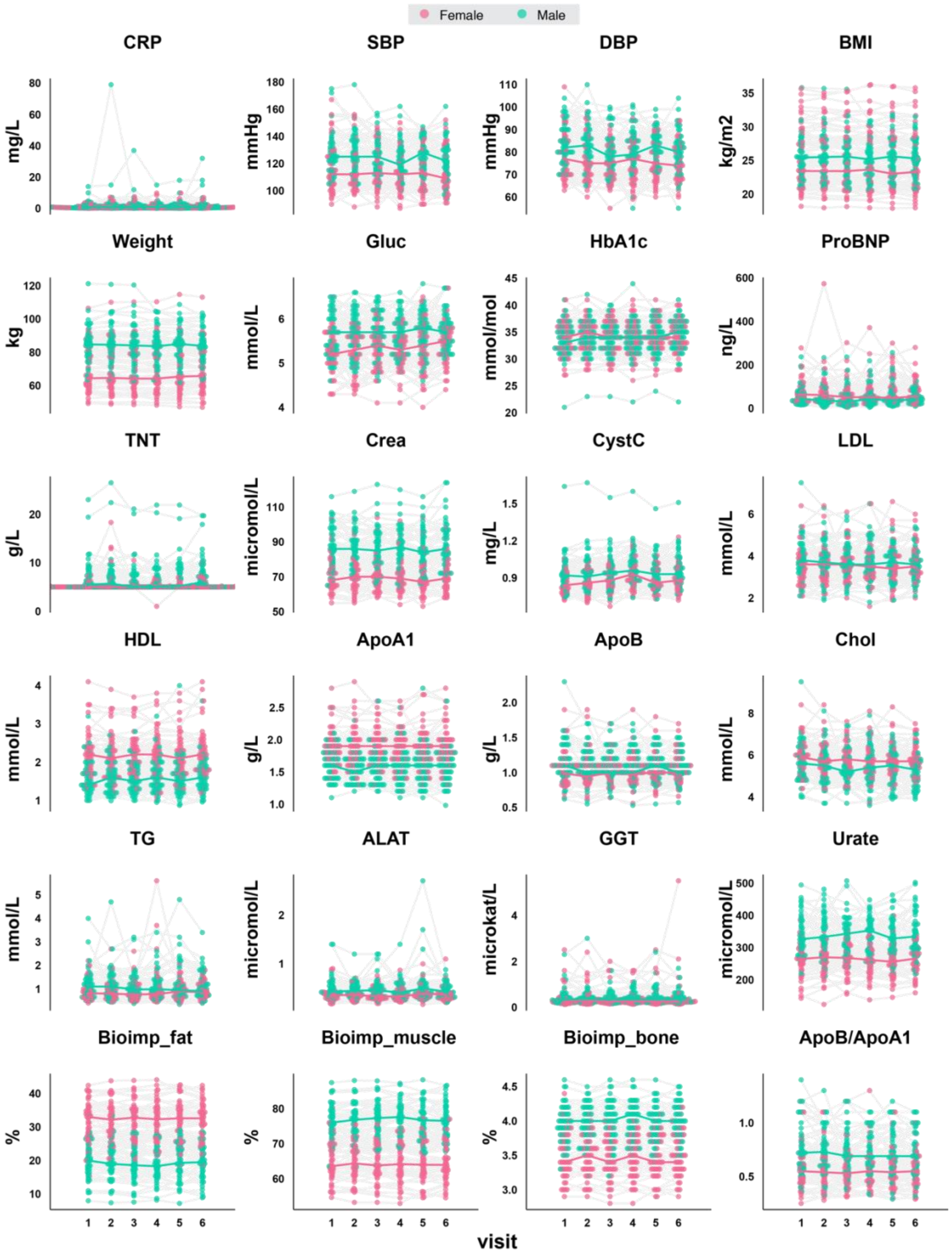

Supplementary Figure 1. **Longitudinal distribution for a selection of clinical parameters**. Longitudinal data across the first six visits for all 94 individuals in a selection of clinical chemistry and hematology variables as well anthropometrics variables. The color indicates males and females, including the medians for each visit and sex, respectively, as colored lines and each individual is connected by a grey line.

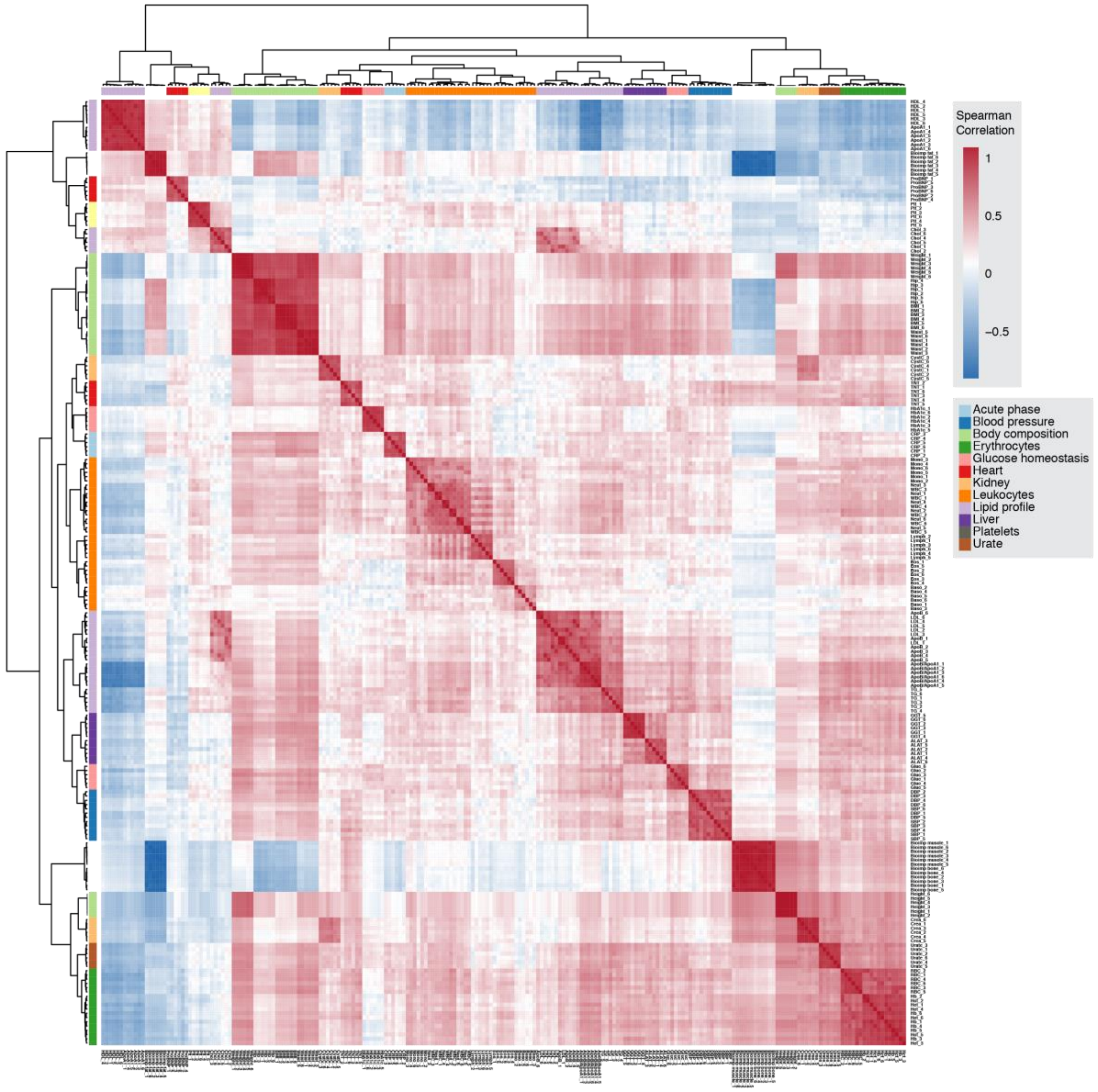

Supplementary Figure 2. Hierarchical clustering of clinical chemistry and hematology variables. Heatmap showing the pairwise Spearman correlation between 37 variables including anthropometrics, clinical chemistry and hematology variables for each of the 94 individuals in visits one to six.

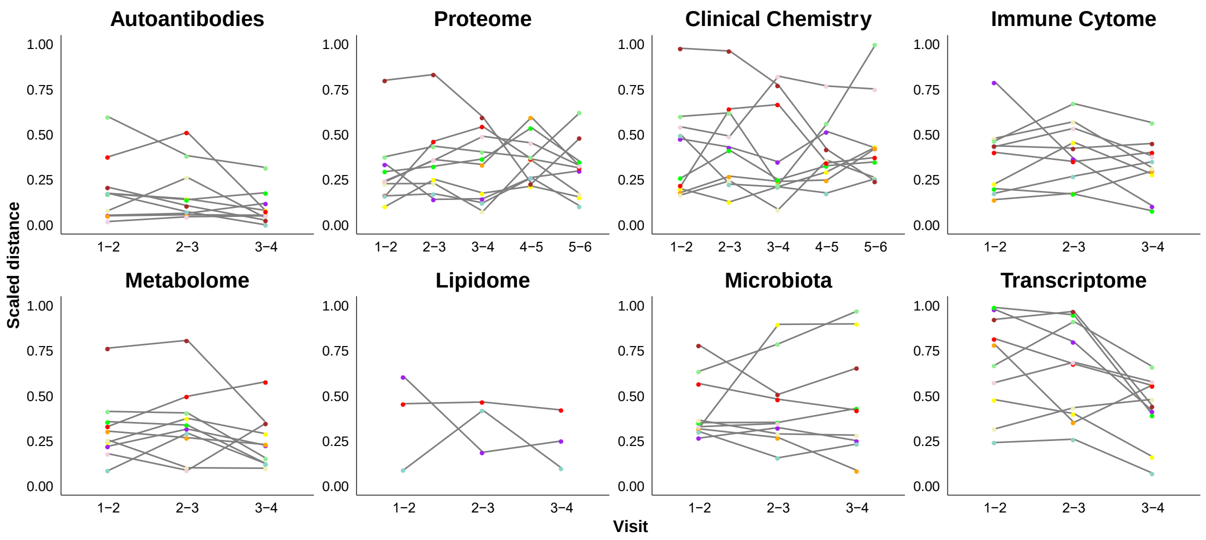

Supplementary Figure 3. Individual inter-visit distance per dataset. Scaled inter-visit distances are based on Euclidian distance (proteome, transcriptome, clinical chemistry, metabolome, lipidome and autoantibodies), Bray-Curtis dissimilarity measure (microbiota) and Aitchinson distance (immune cytome). The ten individuals with the largest average distance based on all datasets are colored separately and the visits of each individual is connected by lines.

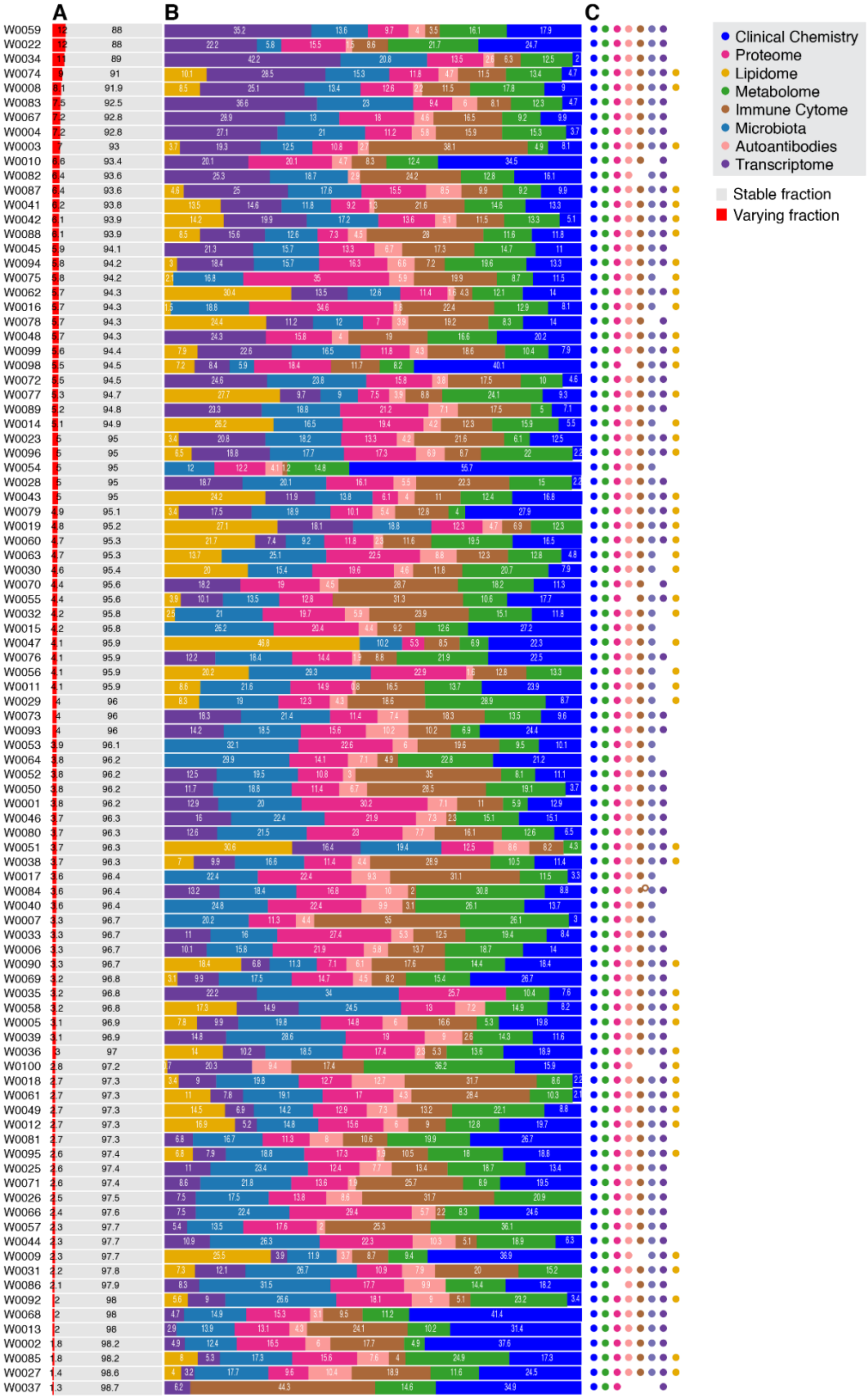

Supplementary Figure 4. Summary of the individual variation. The left column shows the individual identifiers. (**A**) Ranking of individuals based on the "varying fraction" shown in red, calculated as the fraction of features with > 2 SD variation across all data types normalized to the number of features by datasets. (**B**) Contribution of each dataset in the varying fraction shown in A. (**C**) Acquired data types for each individual.

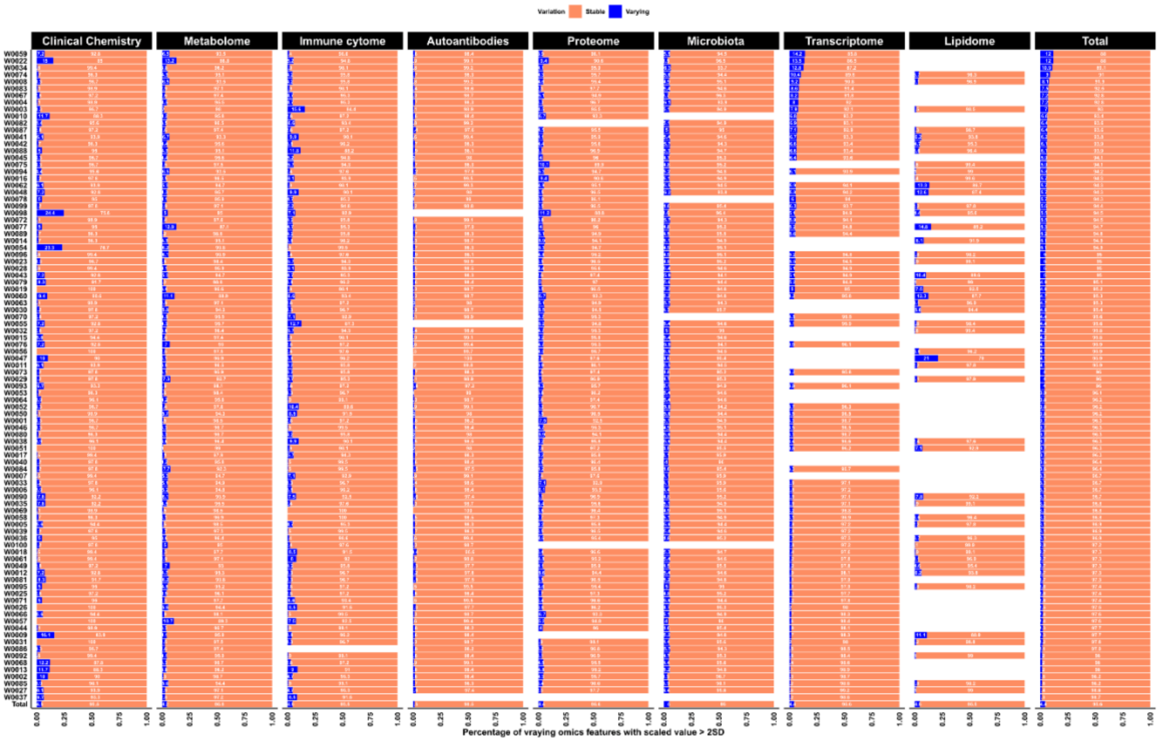

Supplementary Figure 5. Individual variability across datasets. Summary of the varying features per dataset and individual where a feature is considered varying if it has a Z-score > 2 SD. The individuals are sorted according to the "Total" column to the right, summarizing the varying and stable fraction across all datasets. Orange is stable fraction and blue is varying fraction. The bottom row shows a summary based on all individuals per dataset.

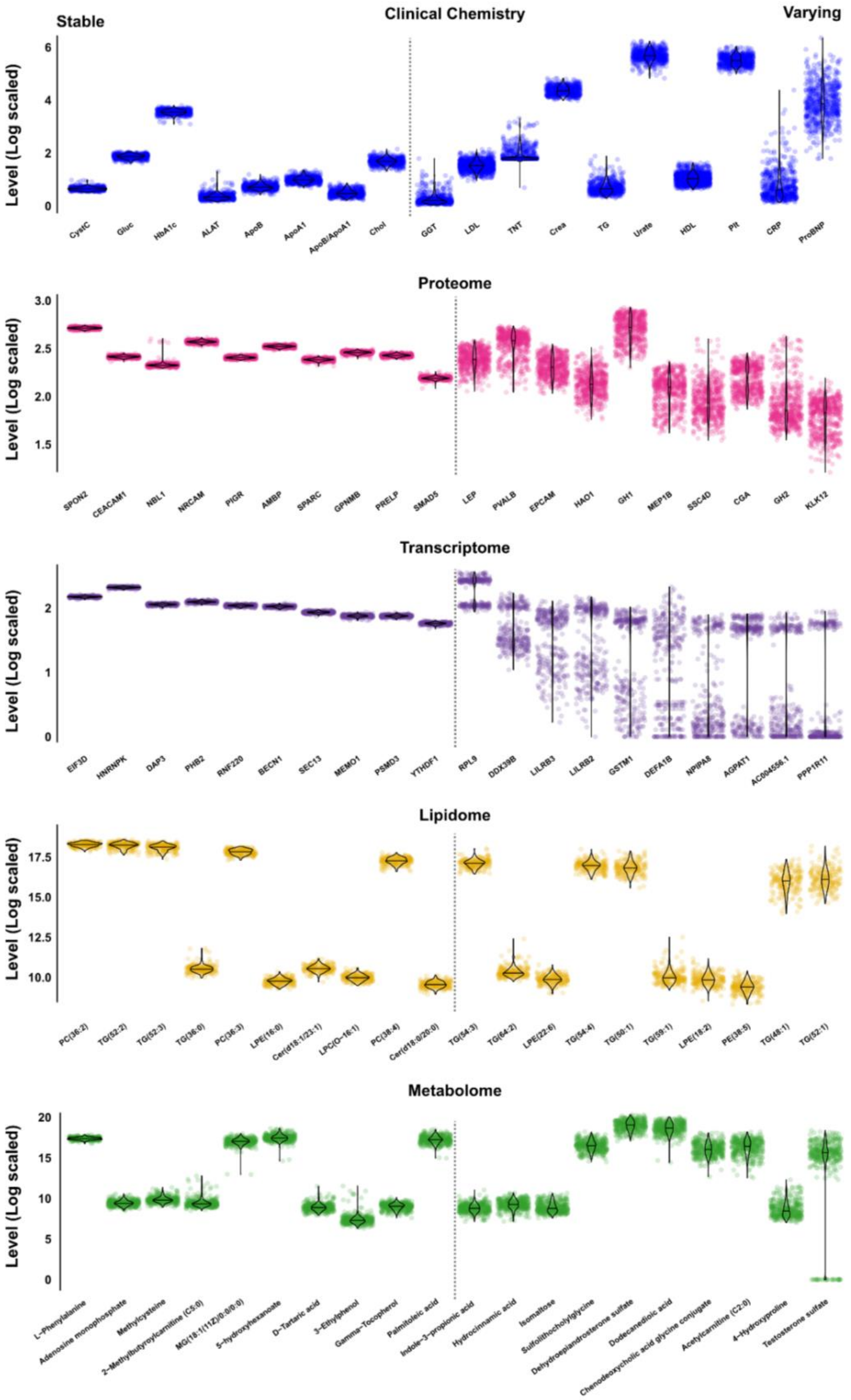

Supplementary Figure 6. Top most stable and most varying variables based on interquartile ranges. (**Left**) Violin plots of top 10 most stable variables, and (**Right**) violin plots of top 10 most varying variables for five of the datasets. Data are represented as violin plots where the middle line is the median.

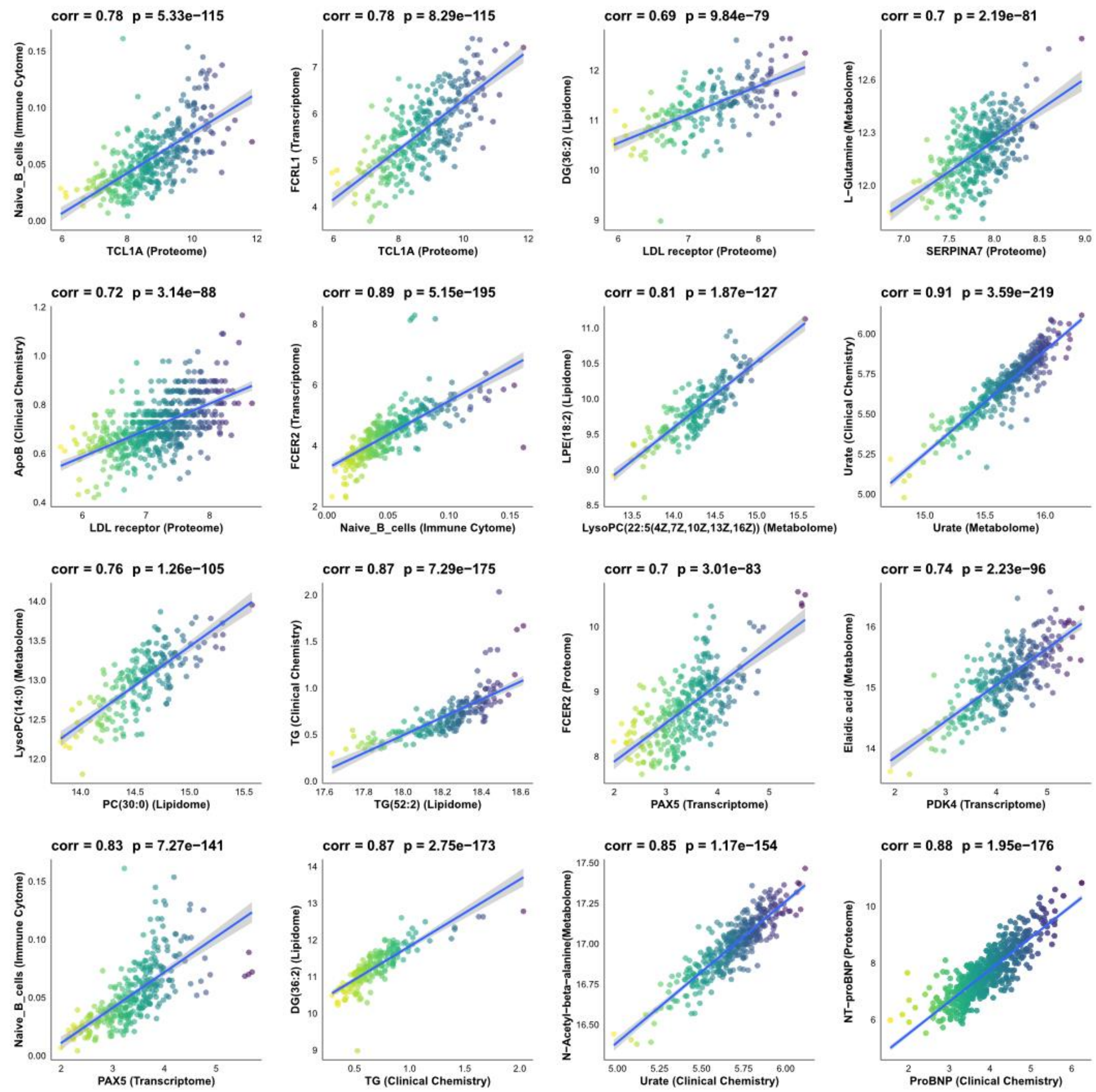

Supplementary Figure 7. Examples of highly correlated variables between datasets. Selected examples with high positive pairwise correlation for two variables from different datasets. Spearman correlation has been used. Multiple test corrections have been applied for *p* values using Benjamini and Hochberg method.

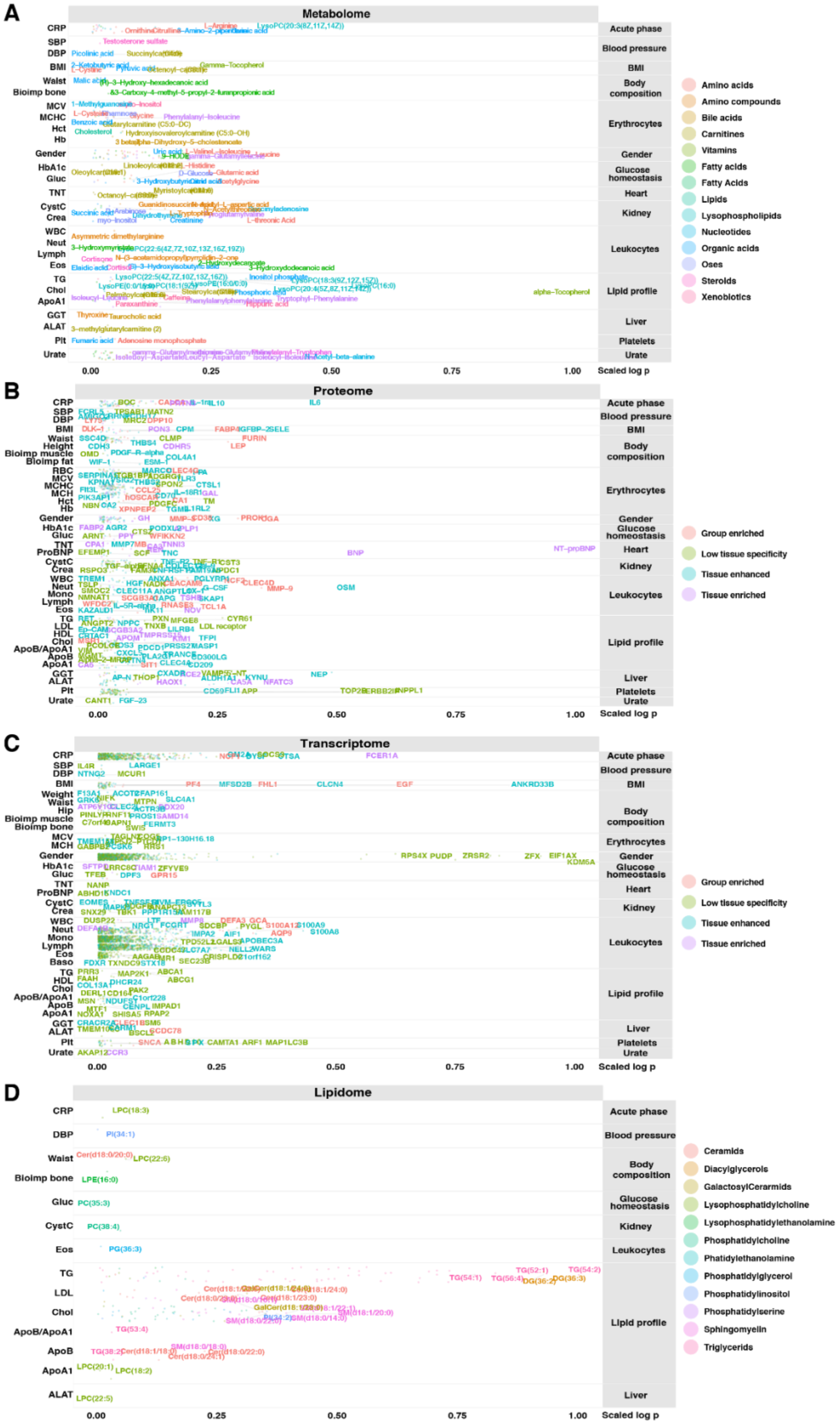

Supplementary Figure 8. Mixed effect results for clinical variables across omics. Most significant variables' effect on clinical parameters for (**A**) metabolome, (**B**) proteome, (**C**) transcriptome and (**D**) lipidome. X-axis shows the scaled and log adjusted p-values.

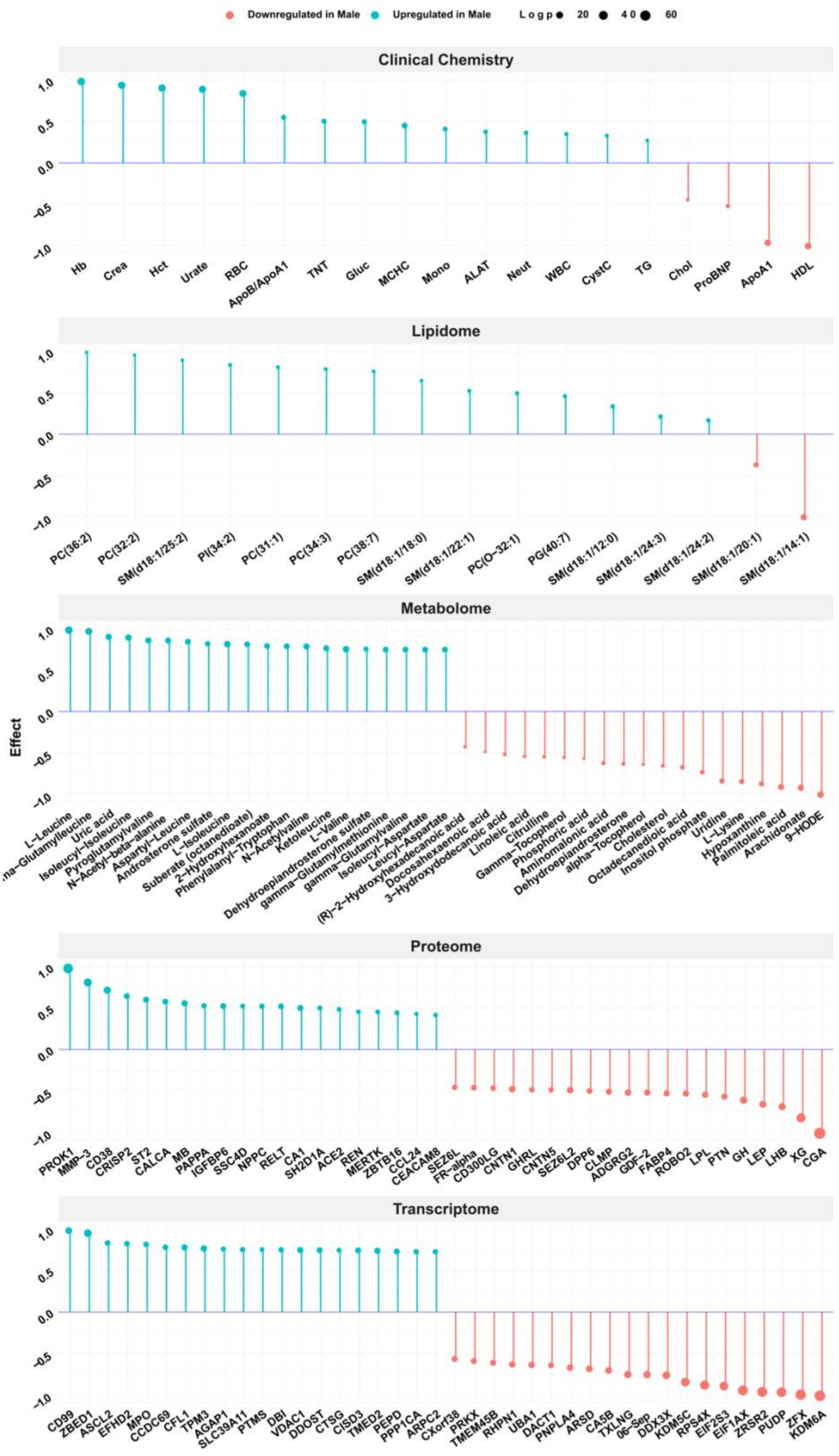

Supplementary Figure 9. Top sex-associated variables retrieved from mixed-effect modeling. Dotplot of the most highly associated variables with sex across significant datasets, with a maximum of top 40 variables.

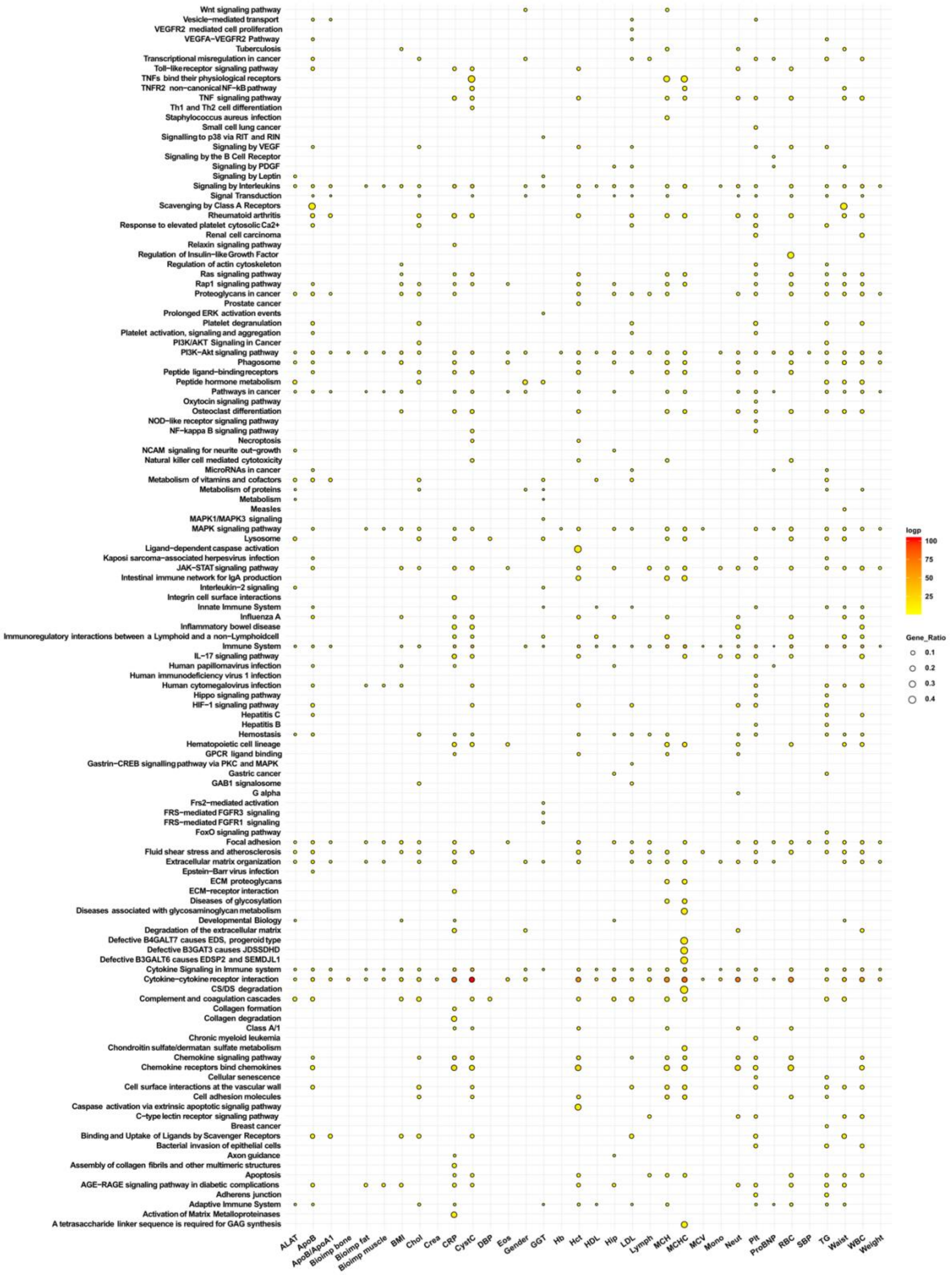

Supplementary Figure 10. **KEGG pathway enrichment analysis of mixed effect modeling results**. Pathways with adjusted p values (Benjamini and Hochberg method) of less than 0.05 are presented. The size of the node represents the ratio of variable-related proteins in specific pathways.
