# Supplementary material for: Integration of molecular profiles in a longitudinal wellness profiling cohort
Source: Nat Commun. 2020 Sep 8;11:4487. doi: 10.1038/s41467-020-18148-7 (PMC7479148; doi:10.1038/s41467-020-18148-7)
Supplement: Supplementary file 7 — Description of Additional Supplementary Files [file 41467_2020_18148_MOESM7_ESM.pdf]

Title: Supplementary Movie 1.
Description: Animation of the longitudinal distribution for a selection of clinical parameters.

Title: Supplementary Movie 2.
Description: Animation of the two dimensional UMAP results.

Title: Supplementary Dataset 1.
Description: Description and summary of the longitudinal clinical data for the 94 subjects that completed the study.

Title: Supplementary Dataset 2.
Description: Complete list of analyzed variables per dataset.

Title: Supplementary Dataset 3.
Description: Complete list of significant mixed effect modelling result.
