# Supplementary material for: Integration of molecular profiles in a longitudinal wellness profiling cohort
Source: Nat Commun. 2020 Sep 8;11:4487. doi: 10.1038/s41467-020-18148-7 (PMC7479148; doi:10.1038/s41467-020-18148-7)
Supplement: Supplementary file 8 — Reporting Summary [file 41467_2020_18148_MOESM8_ESM.pdf]

nature research

Corresponding author(s): Linn Fagerberg

Last updated by author(s): Jul 16, 2020

Reporting Summary

Nature Research wishes to improve the reproducibility of the work that we publish. This form provides structure for consistency and transparency in reporting. For further information on Nature Research policies, see Authors & Referees and the Editorial Policy Checklist .

Statistics

For all statistical analyses, confirm that the following items are present in the figure legend, table legend, main text, or Methods section.

n/a	Confirmed	
☐	☒	The exact sample size (*n*) for each experimental group/condition, given as a discrete number and unit of measurement
☒	☐	A statement on whether measurements were taken from distinct samples or whether the same sample was measured repeatedly
☐	☒	The statistical test(s) used AND whether they are one- or two-sided<br>*Only common tests should be described solely by name; describe more complex techniques in the Methods section.*
☐	☒	A description of all covariates tested
☐	☒	A description of any assumptions or corrections, such as tests of normality and adjustment for multiple comparisons
☐	☒	A full description of the statistical parameters including central tendency (e.g. means) or other basic estimates (e.g. regression coefficient) AND variation (e.g. standard deviation) or associated estimates of uncertainty (e.g. confidence intervals)
☐	☒	For null hypothesis testing, the test statistic (e.g. *F*, *t*, *r*) with confidence intervals, effect sizes, degrees of freedom and *P* value noted<br>*Give P values as exact values whenever suitable.*
☒	☐	For Bayesian analysis, information on the choice of priors and Markov chain Monte Carlo settings
☐	☒	For hierarchical and complex designs, identification of the appropriate level for tests and full reporting of outcomes
☐	☒	Estimates of effect sizes (e.g. Cohen's *d*, Pearson's *r*), indicating how they were calculated

Our web collection on statistics for biologists contains articles on many of the points above.

Software and code

Policy information about availability of computer code

Data collection	16S rRNA gene profiling analysis: PEAR, QIIME (version 1.9.1) , UCLUST , Greengenes reference database, 13_8 release, PyNAST, ChimeraSlayer.<br>Mass spectrometry: Profinder B.08.01<br>RNAseq: Kallisto v.0.43.1
Data analysis	R project for statistical computation; packages: umap, pca, lme4, Enrichr, gglot, gganimate, robCompositions, ICC, pheatmap, tydiverse

For manuscripts utilizing custom algorithms or software that are central to the research but not yet described in published literature, software must be made available to editors/reviewers. We strongly encourage code deposition in a community repository (e.g. GitHub). See the Nature Research guidelines for submitting code & software for further information.

Data

Policy information about availability of data

All manuscripts must include a data availability statement. This statement should provide the following information, where applicable:
- Accession codes, unique identifiers, or web links for publicly available datasets
- A list of figures that have associated raw data
- A description of any restrictions on data availability

The datasets used for this report have been deposited with the Swedish National Data Service (www.snd.se, a data repository certified by Core Trust Seal). The dataset can be made available for validation purposes by contacting snd@snd.gu.se. Data access will be evaluated according to Swedish legislation. Data access for research related questions in the S3WP program can be made available by contacting the corresponding author. All software and code is freely available upon request.

Field-specific reporting

Please select the one below that is the best fit for your research. If you are not sure, read the appropriate sections before making your selection.

- [x] Life sciences
- [ ] Behavioural & social sciences
- [ ] Ecological, evolutionary & environmental sciences

For a reference copy of the document with all sections, see nature.com/documents/nr-reporting-summary-flat.pdf

Life sciences study design

All studies must disclose on these points even when the disclosure is negative.

Sample size	No a priori power calculation was made. The design included the largest possible cohort possible given the high requency of visits and the extensive analyses.
Data exclusions	99 out of 101 invididuals completed the first four visits and 94 the complete 6 visits. For most analyses the individuals that did not complete the first year were excluded. The number of included individuals per dataset has been stated.
Replication	No replication was made.
Randomization	No randomization.
Blinding	No blinding.

Reporting for specific materials, systems and methods

We require information from authors about some types of materials, experimental systems and methods used in many studies. Here, indicate whether each material, system or method listed is relevant to your study. If you are not sure if a list item applies to your research, read the appropriate section before selecting a response.

Materials & experimental systems

n/a	Involved in the study	
☐	☒	Antibodies
☒	☐	Eukaryotic cell lines
☒	☐	Palaeontology
☒	☐	Animals and other organisms
☐	☒	Human research participants
☒	☐	Clinical data

Methods

n/a	Involved in the study	
☒	☐	ChIP-seq
☒	☐	Flow cytometry
☒	☐	MRI-based neuroimaging

Antibodies

Antibodies used	All antibodies used in the study are publicly available through standardized kits provided by Olink Proteomics (www.olink.com)
Validation	The analytical performance of the antibodies have been validated for sensitivity, dynamic range, specificity, precision and scalability, and the results are summarized in the Data Validation documents for each panel, which can be downloaded from the validation data web site (www.olink.com/data-you-can-trust/validation)

Human research participants

Policy information about studies involving human research participants

Population characteristics	Subjects were consecutively recruited from the SCAPIS study. Participants were recruited on the criteria of being clinically healthy and not taking any medication for cardiovascular or pulmonary disease.
Recruitment	Participants were asked by study nurses in the SCAPIS study if they were interested in participating in the S3WP program. Selection as above but no other recruitment bias anticipated.
Ethics oversight	The regional ethical review board in Gothenburg approved the study design.

Note that full information on the approval of the study protocol must also be provided in the manuscript.
